# Supplementary material for: The effect of apathy and compulsivity on planning and stopping in sequential decision-making
Source: PLoS Biol. 2022 Mar 31;20(3):e3001566. doi: 10.1371/journal.pbio.3001566 (PMC8970514; doi:10.1371/journal.pbio.3001566)
Supplement: S1 Text — Contains Supporting information Methods, Results, and Supporting information Tables A–I. Table A: Parameter recovery (schedule used for confirmation sample). The table shows correlations (Pearson’s r) between simulated (“ground truth”) parameters (headers highlighted in grey) and the fitted (“recovered”) parameters (headers with white background) for the decision models of the initial (top) and later (bottom) searches. All parameters showed very good recovery (r > 0.88, diagonal, highlighted in blue). The off-diagonal values show that confusion between parameters was very low (all < 0.22). We used 500 simulated participants for these results. Data in files S13 and S14 Data. Table B: Parameter correlations for initial decisions (confirmation sample). We ran a Pearson correlation of the hierarchically fit parameter estimates from our cognitive model on the initial decisions to look at their relationships. Even though simulations (S1 Table) showed very low parameter confusions, parameters from real participants showed in parts substantial correlations (Pearson’s r), suggesting those correlations are a real relationship existing in participants, rather than an artifact of model fitting. invTemp (inverse temperature of the softmax equation linking value to choice probabilities), SearchBias (bias for or against searching). Prospective (prospective value of the model), Myopic (Myopic value of the model). AvgProspVChange (average change of prospective value per search in the future). Data in file S11 Data. Table C: Parameter correlations for later searches (confirmation sample). We ran a Pearson correlation of the hierarchically fit parameter estimates from our cognitive model on the later decisions to look at their relationships. Even though simulations (S1 Table) showed very low parameter confusions, parameters from real participants showed in parts substantial correlations (Pearson’s r), suggesting those correlations are a real relationship existing in participants, rath [file pbio.3001566.s001.pdf]

Supplements for The effect of apathy and compulsivity on planning and stopping in sequential decision making

Jacqueline Scholl[1,2,3*], Hailey A Trier[3], Matthew F S Rushworth[3,4+], Nils Kolling[4,5+]

[1]Lyon Neuroscience Research Center, INSERM U1028, CNRS UMR5292, PSYR2 Team, University Lyon 1, Lyon, France, [2]Centre Hospitalier Le Vinatier, Pôle EST, F-69500 Bron, France, [3] Wellcome Integrative Neuroimaging (WIN), Department of Experimental Psychology, University of Oxford, Oxford, UK, [4] Centre for Functional MRI of the Brain (FMRIB), Wellcome Integrative Neuroimaging (WIN), Nuffield Department of Clinical Neurosciences, University of Oxford, Oxford, UK, [5]Oxford Centre of Human Brain Activity, Wellcome Integrative Neuroimaging (WIN), Department of Psychiatry, University of Oxford, Oxford, UK, [6]Univ Lyon, Université Lyon 1, Inserm, Stem Cell and Brain Research Institute U1208, Bron, France

[*] Corresponding author: Jacquie.scholl@gmail.com
[+]these authors contributed equally (co-senior author)

SUPPLEMENTARY Tables

Initial decisions

	invTemp	SearchBias	Cost	Myopic	Prospective	AvgProspVCh	invTemp	SearchBias	Cost	Myopic	Prospective	AvgProspVChange
invTemp		0	-0.01	0.02	0	0.01	0.89***	0	0	0.06***	0	0.02
SearchBias	0		0.19***	-0.18***	0.02	0	-0.02	0.99***	0.18***	-0.20***	0.01	0.01
Cost	-0.01	0.19***		0.16***	-0.03*	0	-0.02	0.19***	0.92***	0.15***	-0.03*	-0.01
Myopic	0.02	-0.18***	0.16***		0	0.01	0.03*	-0.19***	0.15***	0.92***	0.01	0.01
Prospective	0	0.02	-0.03*	0		0.01	-0.01	0.02	-0.04**	0.01	0.97***	0.03*
AvgProspVCh	0.01	0	0	0.01	0.01		0.02	0	-0.01	0.01	0.02	0.96***
invTemp	0.89***	-0.02	-0.02	0.03*	-0.01	0.02		-0.03	-0.01	0.07***	-0.01	0.03*
SearchBias	0	0.99***	0.19***	-0.19***	0.02	0	-0.03		0.18***	-0.20***	0.01	0.01
Cost	0	0.18***	0.92***	0.15***	-0.04**	-0.01	-0.01	0.18***		0.17***	-0.04**	0
Myopic	0.06***	-0.20***	0.15***	0.92***	0.01	0.01	0.07***	-0.20***	0.17***		0	0.02
Prospective	0	0.01	-0.03*	0.01	0.97***	0.02	-0.01	0.01	-0.04**	0		0
AvgProspVCh	0.02	0.01	-0.01	0.01	0.03*	0.96***	0.03*	0.01	0	0.02	0	

Later decisions

	invTemp	SearchBias	Cost	Myopic	ProspVS1	ProspVS1mA	PrevSearches	invTemp	SearchBias	Cost	Myopic	ProspVS1	ProspVS1mA	PrevSearches
invTemp		0.01	0.01	0.01	0	-0.02	-0.01	0.85***	0.01	0.02	0.15***	-0.03*	0	-0.02
SearchBias	0.01		-0.01	0.01	-0.02	0	0	0.01	0.97***	-0.05***	0.04**	-0.15***	-0.04**	0.11***
Cost	0.01	-0.01		0.11***	0.09***	-0.09***	0.11***	0.01	0	0.95***	0.12***	0.06***	-0.05***	0.06***
Myopic	0.01	0.01	0.11***		-0.19***	0.19***	-0.18***	-0.02	0	0.09***	0.87***	-0.13***	0.14***	-0.14***
ProspVS1	0	-0.02	0.09***	-0.19***		0.21***	-0.20***	0.02	-0.03*	0.07***	-0.15***	0.88***	0.17***	-0.16***
ProspVS1mA	-0.02	0	-0.09***	0.19***	0.21***		0.16***	0.03*	-0.02	-0.08***	0.14***	0.26***	0.88***	0.13***
PrevSearches	-0.01	0	0.11***	-0.18***	-0.20***	0.16***		0	0	0.11***	-0.17***	-0.14***	0.16***	0.86***
invTemp	0.85***	0.01	0.01	-0.02	0.02	0.03*	0		0.01	0.04**	0.17***	0.01	0.03	-0.06***
SearchBias	0.01	0.97***	0	0	-0.03*	-0.02	0	0.01		-0.04**	0.04**	-0.17***	-0.05***	0.11***
Cost	0.02	-0.05***	0.95***	0.09***	0.07***	-0.08***	0.11***	0.04**	-0.04**		0.12***	0.09***	-0.03*	0.04**
Myopic	0.15***	0.04**	0.12***	0.87***	-0.15***	0.14***	-0.17***	0.17***	0.04**	0.12***		-0.12***	0.15***	-0.18***
ProspVS1	-0.03*	-0.15***	0.06***	-0.13***	0.88***	0.26***	-0.14***	0.01	-0.17***	0.09***	-0.12***		0.25***	-0.18***
ProspVS1mA	0	-0.04**	-0.05***	0.14***	0.17***	0.88***	0.16***	0.03	-0.05***	-0.03*	0.15***	0.25***		0.07***
PrevSearches	-0.02	0.11***	0.06***	-0.14***	-0.16***	0.13***	0.86***	-0.06***	0.11***	0.04**	-0.18***	-0.18***	0.07***	

Table A. Parameter recovery (schedule used for confirmation sample). The table shows correlations (Pearson's r) between simulated ('ground truth') parameters (headers highlighted in grey) and the fitted ('recovered') parameters (headers with white background) for the decision models of the initial (top) and later (bottom) searches. All parameters showed very good recovery (r>0.88, diagonal, highlighted in blue). The off-diagonal values show that confusion between parameters was very low (all < 0.22). We used 500 simulated participants for these results. Data in files 13 and 14.

	invTemp	*Offer*	*SearchBias*	*Cost*	*Prospective*	*Myopic*	*AvgProspVChange*
invTemp		0.30^{***}	0.26^{***}	0.04	0.30^{***}	-0.21^{***}	0.30^{***}
Offer	0.30^{***}		0.82^{***}	0.17^{***}	0.42^{***}	-0.65^{***}	0.44^{***}
SearchBias	0.26^{***}	0.82^{***}		0.53^{***}	0.26^{***}	-0.69^{***}	0.40^{***}
Cost	0.04	0.17^{***}	0.53^{***}		0.08^{*}	-0.22^{***}	0.02
Prospective	0.30^{***}	0.42^{***}	0.26^{***}	0.08^{*}		-0.54^{***}	0.61^{***}
Myopic	-0.21^{***}	-0.65^{***}	-0.69^{***}	-0.22^{***}	-0.54^{***}		-0.53^{***}
AvgProspVChange	0.30^{***}	0.44^{***}	0.40^{***}	0.02	0.61^{***}	-0.53^{***}	

Computed correlation used pearson-method with listwise-deletion.

Table B. Parameter correlations for initial decisions (confirmation sample). We ran a pearson correlation of the hierarchically fit parameter estimates from our cognitive model on the initial decisions to look at their relationships. Even though simulations (table S1) showed very low parameter confusions, parameters from real participants showed in parts substantial correlations (Pearson's r), suggesting those correlations are a real relationship existing in participants, rather than an artifact of model fitting. invTemp (inverse temperature of the softmax equation linking value to choice probabilities), SearchBias (bias for or against searching). Prospective (prospective value of the model), Myopic (Myopic value of the model). AvgProspVChange (average change of prospective value per search in the future). Data in file 11.

	invTemp	*SearchBias*	*Cost*	*Offer*	*Myopic*	*ProspVS1*	*ProspVS1mAdapted*	*PrevSearches*	*TotalCost*
invTemp		0.38^{***}	0.05	-0.08^{*}	0.21^{***}	0.23^{***}	-0.19^{***}	-0.37^{***}	-0.07
SearchBias	0.38^{***}		0.37^{***}	-0.39^{***}	0.44^{***}	0.62^{***}	-0.23^{***}	-0.31^{***}	0.05
Cost	0.05	0.37^{***}		-0.41^{***}	0.35^{***}	0.20^{***}	-0.18^{***}	-0.10^{**}	0.29^{***}
Offer	-0.08^{*}	-0.39^{***}	-0.41^{***}		-0.82^{***}	0.05	0.01	-0.01	-0.13^{***}
Myopic	0.21^{***}	0.44^{***}	0.35^{***}	-0.82^{***}		-0.03	-0.09^{*}	-0.12^{***}	0.02
ProspVS1	0.23^{***}	0.62^{***}	0.20^{***}	0.05	-0.03		-0.20^{***}	-0.30^{***}	-0.03
ProspVS1mAdapted	-0.19^{***}	-0.23^{***}	-0.18^{***}	0.01	-0.09^{*}	-0.20^{***}		0.33^{***}	0.04
PrevSearches	-0.37^{***}	-0.31^{***}	-0.10^{**}	-0.01	-0.12^{***}	-0.30^{***}	0.33^{***}		0.46^{***}
TotalCost	-0.07	0.05	0.29^{***}	-0.13^{***}	0.02	-0.03	0.04	0.46^{***}	

Computed correlation used pearson-method with listwise-deletion.

Table C: **Parameter correlations for later searches** (confirmation sample). We ran a pearson correlation of the hierarchically fit parameter estimates from our cognitive model on the later decisions to look at their relationships. Even though simulations (table S1) showed very low parameter confusions, parameters from real participants showed in parts substantial correlations (Pearson's r), suggesting those correlations are a real relationship existing in participants, rather than an artifact of model fitting. Abbreviations same as Table S2. ProspVS1 (prospective value at first search).

ProspVS1mAdapted (change of prospective value since first search). PrevSearches (number of previous searches). Total Cost (sum of total cost since first search). Data in file 9.

	Excl. not enough variation in later decision responses N=39	Excl. questionnaires N=2	Excl. task understanding N=33	N=756	p-value
Depression (score)	17.5 (13.7)	6.00 (2.83)	16.6 (13.0)	14.6 (11.8)	0.263
Apathy (score)	26.6 (7.20)	17.0 (1.41)	26.5 (10.2)	29.7 (8.72)	0.005
Obsession-compulsion (score)	24.5 (15.3)	13.0 (2.83)	26.5 (12.2)	20.4 (12.8)	0.011
Social anxiety (score)	52.4 (34.7)	. (.)	59.2 (32.7)	53.2 (31.0)	0.554
Alexithymia (score)	45.2 (9.59)	35.0 (4.24)	44.4 (9.11)	43.2 (9.67)	0.329
Fatigue (score)	26.6 (9.35)	15.0 (0.00)	25.9 (9.32)	25.4 (8.70)	0.313
Trait anxiety (score)	46.6 (12.9)	28.0 (5.66)	47.3 (13.3)	46.1 (13.2)	0.255
Depression (categorical):					.
Minimal	19 (48.7%)	2 (100%)	15 (45.5%)	392 (51.9%)	
Mild	4 (10.3%)	0 (0.00%)	7 (21.2%)	136 (18.0%)	
Moderate	8 (20.5%)	0 (0.00%)	5 (15.2%)	114 (15.1%)	
Severe	8 (20.5%)	0 (0.00%)	6 (18.2%)	114 (15.1%)	
Obsession-compulsion (categorical):					0.029
None	16 (41.0%)	2 (100%)	13 (39.4%)	426 (56.3%)	
Clinically significant OC	23 (59.0%)	0 (0.00%)	20 (60.6%)	330 (43.7%)	
Social anxiety (categorical):					0.525
None	10 (25.6%)	0 (0.00%)	7 (21.2%)	202 (26.7%)	
Social anx. possible	11 (28.2%)	0 (0.00%)	7 (21.2%)	238 (31.5%)	
Social anx. probable	18 (46.2%)	2 (100%)	19 (57.6%)	316 (41.8%)	
Alexithymia (categorical):					0.387
None	27 (69.2%)	2 (100%)	26 (78.8%)	607 (80.3%)	
Alexithymia possible	9 (23.1%)	0 (0.00%)	7 (21.2%)	120 (15.9%)	
Alexithymia present	3 (7.69%)	0 (0.00%)	0 (0.00%)	29 (3.84%)	
Fatigue (categorical):					0.832
None	14 (35.9%)	2 (100%)	12 (36.4%)	290 (38.4%)	
Present	16 (41.0%)	0 (0.00%)	14 (42.4%)	326 (43.1%)	

Extreme fatigue	9 (23.1%)	0 (0.00%)	7 (21.2%)	140 (18.5%)

Table D: Additional demographics (confirmation sample). Participants completed the following questionnaires. Apathy Motivation Index [1] (AMI), Obsessive Compulsive Inventory [2] (OCI-R), Beck's depression inventory [3] (BDI), Spielberger State Anxiety [4], Liebowitz Social anxiety [5], Short Scales for Measuring Schizotypy [6], ('unusual experiences' and 'introvertive anhedonia' subscale), Fatigue scale [7], Toronto alexithymia scale [8]. Depression cut offs were (less than 13 'Minimal', less then 20 'Mild', less than 29 'Moderate' and higher or equal 29 'Severe'). Obsessive-compulsion less than 21 'none', otherwise 'clinically significant'). For social anxiety we had less than 30 as 'none', between 30 but less than 60 as 'possible' and at and above 60 as 'probable' social anxiety. For Alexithymia we had less than 52 as none, between 52 but less than 61 as 'possible' and at and above 62 as 'present' Alexithymia. Fatigue was 'none' below 22, 'present' between 22 and 34 and 'extreme' above 34. Data in file 5.

Confirmation sample. controlled for medication status

	**Over-pers. [dec, later]**		**Cost insensitivity [dec, later]**		**Chasing [self rep]**		**Preempt. avoid [self rep]**	
Predictors	*Estimates*	*CI (90%)*	*Estimates*	*CI (90%)*	*Odds Ratios*	*CI (90%)*	*Odds Ratios*	*CI (90%)*
Compulsivity	0.05	-0.01 – 0.12	0.06	-0.01 – 0.13	1.22	1.08 – 1.38	1.42	1.25 – 1.61
Apathy	0.07	0.01 – 0.13	0.01	-0.06 – 0.07	0.89	0.79 – 1.00	0.75	0.66 – 0.84
DeprAnx	-0.00	-0.08 – 0.08	-0.02	-0.11 – 0.06	0.99	0.84 – 1.15	1.02	0.87 – 1.20
SocialAnx	-0.04	-0.12 – 0.04	0.03	-0.06 – 0.11	1.24	1.06 – 1.43	1.26	1.09 – 1.47
Age	0.05	-0.01 – 0.10	0.09	0.02 – 0.15	0.87	0.77 – 0.98	0.79	0.70 – 0.89
Gender: Male	0.05	-0.08 – 0.17	0.02	-0.11 – 0.15	0.73	0.58 – 0.92	1.31	1.03 – 1.65
Medication status	0.07	-0.13 – 0.27	0.09	-0.13 – 0.30	0.89	0.61 – 1.30	0.79	0.52 – 1.18
Education	-0.02	-0.09 – 0.05	0.02	-0.06 – 0.09	0.97	0.83 – 1.11	1.06	0.92 – 1.20
Observations	753		753		753		753	
R^2 Bayes	0.152		0.031		0.087		0.109	

Table E. Results of regressions linking clinical dimensions to decision and self-report measures controlling for medication status (confirmation sample). As control regressors, we included age, gender and education (as in main manuscript). Additionally, here, psychoactive medication status is also included (coded as 'Yes'/'No' as there were too few respondents of individual types of medications). Data in files2, 6, 9 and 16.

Later searches [decision], Confirmation sample

	Over-pers.		InvTemp		Offer		Cost	
Predictors	*Estimates*	*CI (90%)*	*Estimates*	*CI (90%)*	*Estimates*	*CI (90%)*	*Estimates*	*CI (90%)*
Compulsivity	0.053	-0.010 – 0.117	-0.149	-0.216 – -0.082	0.056	-0.011 – 0.124	0.061	-0.006 – 0.129
Apathy	0.066	0.005 – 0.125	0.007	-0.056 – 0.071	0.026	-0.039 – 0.090	0.004	-0.061 – 0.070
DeprAnx	0.005	-0.073 – 0.081	0.093	0.012 – 0.174	-0.110	-0.194 – -0.029	-0.017	-0.101 – 0.066
SocialAnx	-0.040	-0.114 – 0.037	-0.062	-0.144 – 0.019	0.107	0.025 – 0.189	0.027	-0.055 – 0.110
Age	0.046	-0.014 – 0.107	0.059	-0.003 – 0.122	-0.066	-0.130 – -0.002	0.088	0.024 – 0.152
Gender: Male	0.045	-0.076 – 0.165	0.234	0.110 – 0.361	-0.144	-0.272 – -0.016	0.016	-0.113 – 0.144
bsp_moEducation	-0.018	-0.090 – 0.055	-0.003	-0.080 – 0.072	0.034	-0.040 – 0.127	0.024	-0.056 – 0.094

	CostxPrevSearch		Prospective		ProspValDiff		Myopic		ForageBias	
	Estimates	*CI (90%)*	*Estimates*	*CI (90%)*	*Estimates*	*CI (90%)*	*Estimates*	*CI (90%)*	*Estimates*	*CI (90%)*
Compulsivity	0.059	-0.009 – 0.127	-0.061	-0.126 – 0.007	0.051	-0.016 – 0.119	-0.063	-0.131 – 0.006	-0.016	-0.080 – 0.048
Apathy	0.023	-0.042 – 0.088	0.006	-0.057 – 0.070	0.014	-0.050 – 0.077	-0.019	-0.082 – 0.045	-0.003	-0.063 – 0.057
DeprAnx	0.000	-0.083 – 0.085	0.012	-0.070 – 0.092	0.022	-0.059 – 0.106	0.097	0.015 – 0.179	0.053	-0.025 – 0.132
SocialAnx	-0.038	-0.122 – 0.045	0.039	-0.043 – 0.121	0.022	-0.059 – 0.104	-0.072	-0.153 – 0.010	-0.038	-0.115 – 0.039
Age	0.076	0.011 – 0.140	-0.065	-0.128 – -0.004	0.022	-0.041 – 0.087	0.041	-0.022 – 0.103	-0.037	-0.096 – 0.024
Gender: Male	0.146	0.014 – 0.278	0.109	-0.018 – 0.238	0.104	-0.022 – 0.230	0.092	-0.035 – 0.223	0.057	-0.065 – 0.174
bsp_moEducat	0.003	-0.088 – 0.082	-0.003	-0.087 – 0.071	-0.086	-0.159 – -0.021	0.021	-0.050 – 0.096	0.004	-0.073 – 0.077

Table F. Results of regressions linking clinical dimensions to later search decisions. *Results of all parameters from model fit to the later search decisions and dimensions. We used 90% two-sided Confidence intervals (CI), to show the results of one-sided (95% CI) tests for our pre-registered hypotheses and to show what other links have any evidence. Data in files 9 and 16.*

First search [decision], Confirmation sample

	Pre-empt avoid.[rev]		InvTemp		Offer		Cost	
Predictors	*Estimates*	*CI (90%)*	*Estimates*	*CI (90%)*	*Estimates*	*CI (90%)*	*Estimates*	*CI (90%)*
Compulsivity	0.044	-0.021 – 0.110	-0.206	-0.273 – -0.138	-0.017	-0.084 – 0.049	0.082	0.013 – 0.151
Apathy	0.003	-0.062 – 0.067	-0.032	-0.098 – 0.034	0.020	-0.044 – 0.084	-0.041	-0.107 – 0.027
DeprAnx	-0.023	-0.104 – 0.058	0.091	0.009 – 0.173	0.050	-0.032 – 0.132	0.058	-0.025 – 0.143
SocialAnx	-0.009	-0.087 – 0.071	-0.018	-0.100 – 0.062	-0.003	-0.082 – 0.074	-0.108	-0.190 – -0.026
Age	0.033	-0.030 – 0.097	0.063	-0.002 – 0.127	-0.125	-0.188 – -0.061	0.039	-0.028 – 0.106
Gender: Male	-0.219	-0.348 – -0.094	0.248	0.122 – 0.375	0.050	-0.074 – 0.179	0.074	-0.055 – 0.206
bsp_moEducation	0.100	0.019 – 0.205	-0.036	-0.138 – 0.036	0.081	0.015 – 0.149	0.035	-0.038 – 0.108

	Prospective		Myopic		ForageBias	
	Estimates	*CI (90%)*	*Estimates*	*CI (90%)*	*Estimates*	*CI (90%)*
Compulsivity	-0.052	-0.119 – 0.013	-0.020	-0.089 – 0.049	-0.004	-0.072 – 0.065
Apathy	0.025	-0.039 – 0.090	-0.059	-0.125 – 0.006	-0.007	-0.072 – 0.058
DeprAnx	-0.038	-0.120 – 0.045	-0.083	-0.166 – -0.002	0.083	0.001 – 0.166
SocialAnx	0.025	-0.055 – 0.104	0.087	0.007 – 0.167	-0.048	-0.126 – 0.031
Age	-0.071	-0.134 – -0.007	0.117	0.053 – 0.182	-0.064	-0.127 – 0.001
Gender: Male	0.174	0.049 – 0.303	-0.109	-0.239 – 0.021	0.090	-0.038 – 0.221
bsp_moEducation	0.040	-0.034 – 0.145	0.016	-0.058 – 0.117	0.038	-0.050 – 0.105

Table G. Results of regressions linking clinical dimensions to initial search decisions. *Results of all parameters from model fit to the initial search decisions and dimensions. We used 90% two-sided Confidence intervals (CI), to show the results of one-sided tests for our pre-registered hypotheses and to show what other links have any evidence. Data in files 11 and 16.*

Self report, Confirmation sample

	Pre-empt. avoid		Aware decision inertia		Chasing		Cost insensitiv	
Predictors	*Odds Ratios*	*CI (90%)*	*Odds Ratios*	*CI (90%)*	*Odds Ratios*	*CI (90%)*	*Odds Ratios*	*CI (90%)*
Compulsivity	1.423	1.255 – 1.613	1.608	1.411 – 1.830	1.224	1.081 – 1.384	1.292	1.144 – 1.462
Apathy	0.753	0.667 – 0.850	0.882	0.778 – 0.999	0.891	0.793 – 1.003	0.896	0.798 – 1.006
DeprAnx	0.999	0.858 – 1.169	0.945	0.810 – 1.102	0.974	0.838 – 1.131	1.192	1.024 – 1.386
SocialAnx	1.256	1.079 – 1.467	1.082	0.925 – 1.266	1.234	1.064 – 1.432	1.149	0.989 – 1.332
Age	0.789	0.700 – 0.886	0.928	0.824 – 1.046	0.867	0.771 – 0.972	0.939	0.837 – 1.055
Gender: Male	1.301	1.026 – 1.646	1.038	0.813 – 1.323	0.731	0.580 – 0.925	0.820	0.646 – 1.036
bsp_moEducation	1.060	0.921 – 1.201	0.908	0.784 – 1.031	0.973	0.824 – 1.101	0.904	0.762 – 1.032

Table H. Results of regressions linking clinical dimensions to self-report measures. *Results of all self-report measures fit to the dimensions. We used 90% two-sided Confidence intervals (CI), to show the results of one-sided tests for our pre-registered hypotheses and to show what other links have any evidence. Data in files 2 and 16.*

	**Excl. not enough variation in later decision responses N=39**	**Excl. questionnaires N=2**	**Excl. task understanding N=33**	**Included N=756**	**p-value**
Selective serotonin reuptake inhibitor:					0.53
NO	37 (94.9%)	2 (100%)	33 (100%)	709 (93.8%)	
Yes	2 (5.13%)	0 (0.00%)	0 (0.00%)	47 (6.22%)	
Serotonin-noradrenaline reuptake inhibitor:					1
NO	39 (100%)	2 (100%)	33 (100%)	744 (98.4%)	
Yes	0 (0.00%)	0 (0.00%)	0 (0.00%)	12 (1.59%)	
Serotonin [mixed]:					1
NO	39 (100%)	2 (100%)	33 (100%)	754 (99.7%)	
Yes	0 (0.00%)	0 (0.00%)	0 (0.00%)	2 (0.26%)	
Tricylic antidepressant:					1
NO	39 (100%)	2 (100%)	33 (100%)	755 (99.9%)	
Yes	0 (0.00%)	0 (0.00%)	0 (0.00%)	1 (0.13%)	
Tetracyclic antidepressant:					1
NO	39 (100%)	2 (100%)	33 (100%)	754 (99.7%)	
Yes	0 (0.00%)	0 (0.00%)	0 (0.00%)	2 (0.26%)	
Serotonin agonist:					1
NO	39 (100%)	2 (100%)	33 (100%)	755 (99.9%)	
Yes	0 (0.00%)	0 (0.00%)	0 (0.00%)	1 (0.13%)	
Serotonin [atypical]:					1
NO	39 (100%)	2 (100%)	33 (100%)	755 (99.9%)	
Yes	0 (0.00%)	0 (0.00%)	0 (0.00%)	1 (0.13%)	
Melatonin:					1

NO	39 (100%)	2 (100%)	33 (100%)	755 (99.9%)	
Yes	0 (0.00%)	0 (0.00%)	0 (0.00%)	1 (0.13%)	
Serotonin precursor:					1
NO	39 (100%)	2 (100%)	33 (100%)	755 (99.9%)	
Yes	0 (0.00%)	0 (0.00%)	0 (0.00%)	1 (0.13%)	
Atypical antipsychotic:					0.48
NO	38 (97.4%)	2 (100%)	33 (100%)	750 (99.2%)	
Yes	1 (2.56%)	0 (0.00%)	0 (0.00%)	6 (0.79%)	
Cannabis:					1
NO	39 (100%)	2 (100%)	33 (100%)	753 (99.6%)	
Yes	0 (0.00%)	0 (0.00%)	0 (0.00%)	3 (0.40%)	
Psychedelic:					0
NO	39 (100%)	1 (50.0%)	33 (100%)	756 (100%)	
Yes	0 (0.00%)	1 (50.0%)	0 (0.00%)	0 (0.00%)	
Beta blocker:					1
NO	39 (100%)	2 (100%)	33 (100%)	754 (99.7%)	
Yes	0 (0.00%)	0 (0.00%)	0 (0.00%)	2 (0.26%)	
Benzodiazepine:					1
NO	39 (100%)	2 (100%)	33 (100%)	754 (99.7%)	
Yes	0 (0.00%)	0 (0.00%)	0 (0.00%)	2 (0.26%)	
Sodium channel blocker/ anticonvulsant:					1
NO	39 (100%)	2 (100%)	33 (100%)	754 (99.7%)	
Yes	0 (0.00%)	0 (0.00%)	0 (0.00%)	2 (0.26%)	
Noradrenaline/dopamine reuptake inhibitor:					1
NO	39 (100%)	2 (100%)	33 (100%)	751 (99.3%)	
Yes	0 (0.00%)	0 (0.00%)	0 (0.00%)	5 (0.66%)	
Sedating antihistamine:					1
NO	39 (100%)	2 (100%)	33 (100%)	755 (99.9%)	
Yes	0 (0.00%)	0 (0.00%)	0 (0.00%)	1 (0.13%)	

Table I: Additional medication information (confirmation sample). All information for the types of medications excluded and included participants used (NO and Yes). The most common medication were selective serotonin reuptake inhibitor. Data in file 6.

SUPPLEMENTARY METHODS

Decision tree model to derive prospective value

As the decision tree model is identical to a previous paper [9] we include the model description from there:

“

The aim of the model is to compute the value of searching taking into account the possibility for future searches given the current search horizon (available number of searches), the alternatives' properties, and search costs. This model-based value can then be compared to the current offer to make the decision of whether to accept the offer or to search for a better alternative. To find the value of searching, the model simulates a series of full decision trees, each time applying a different acceptance-threshold, i.e. a value above which it would accept a new offer and otherwise reject it to search again. Examples of decision trees are shown in figure S1. For each end-state of the decision tree (i.e. 'branch'), the model computes the probability of this state given the acceptance-threshold. It then multiplies this probability with the reward value of the end-state outcome (taking into account both the reward received and the cost of the number of searches taken to get to this point). The value of searching (VSearch) thus becomes:

$$VSearch = \sum_{s=1}^{maxSearch-1} [p(Search)^{s-1} * (AcceptUtility - s * cost * p(Accept)] + p(Search)^{maxSearch-1} * (Myopic\ utility - maxSearch * cost)$$

Where p(Search) is the probability of searching which is equivalent to the summed probabilities of the alternatives below a given acceptance threshold. P(Accept) is the opposite: the probability of not searching and instead accepting an alternative in the decision tree (i.e. an offer that is received). MaxSearches corresponds to the search horizon: the maximum number of searches possible in a trial and therefore the depth of the decision tree. Cost is the point loss incurred for every search decision on a given trial […]. Note, the model also employs a changing acceptance-thresholds at each search step; it allows for different p(Search)and p(Accept) for every step in the sequence (or level of the tree; fig.S1B). The term s corresponds to the search number within the tree (e.g. s=1 is the first search in an environment). " [9].
See also the previous paper [9], supplements figure S1 for a visualization of the model.

Task schedule design and model validation
When designing the task schedules for the confirmation sample, we ensured that it would allow us to reliably measure participants' behaviour. Specifically, this was achieved by generating random schedules (given the constraint for variables such as number of trials and levels of cost as detailed in the methods of the main text). We then simulated choices of 500 participants given random combinations of model parameters within the ranges of those found for the real participants of the discovery sample.

We then analysed these simulated choices with the same decision models as for the real participants. The only difference was that we did not use a hierarchical model and that the model was fit using Stan's Variational Bayes algorithm, rather than sampling to increase speed of the procedure. The reason we used a non-hierarchical model to fit the simulated data was because parameter combinations were drawn with a flat distribution in the ranges of real participants, rather than following a normal distribution. We then checked, for both types of analyses (i.e. first choice and later choices on each trial separately) that the correlations between the simulated ('ground truth') and the fitted parameters were r>0.7 (see figure S5). If a schedule did not fulfil these criteria it was abandoned and a new schedule was created.

As a technical aside, we found that the most reliable way to simulate the data was to z-score the variables before scaling them by each simulated subject's parameters – for the analysis of the first search – exactly as was done with the real data. For the analysis of the later searches this was not possible as what offers simulated participants would encounter would depend on their choices (e.g. if they did not search on search 1 of trial 1, there would of course be no 'later searches' for that trial). Therefore, we instead standardized the variables using the mean and standard deviation from the discovery sample. The checks of the correlations between simulated and real described above only ensure consistency of the parameter values within a given schedule. It is possible that across schedules there is no such consistency; for example, let's assume a simulated parameter is in the range 0 to 1 and in schedule A recovers with r=0.8 in the range 0 to 1 and in schedules B recovers with r=0.8 in the range of 1 to 2. Then the overall parameter recovery across schedules would be bad. Thus, we combined data from all simulated participants across all schedules and checked the correlations, again as r>0.7.

To check how well our decision model fitted our data, we computed the negative log likelihood for each measurement from each participant using a half-split cross-validation procedure. Specifically, for each participant we selected a random half of their data points and then fitted hierarchical models to these 'training data'. We then applied the parameters to the 'test data' (remaining half of data) and computed for each data point the fit (negative log likelihood). As a cross-validation procedure was used, no further adjustment for the number of model parameters was needed. We compared models that contained all regressors of interest, as well as control regressors (see figure S2A), with models where iteratively one of the regressors was removed (figure S2C). The aim was to test how important each of the regressors was for capturing participants' behaviour. We compared the model fit by computing the sum of the negative log likelihoods across participants, lower values suggest better fit.

SUPPLEMENTARY RESULTS

We analysed the behavioural data with cognitive models based on our previous paper [9]. This was done separately for the initial search decision on each trial and for the later decisions on each trial. In short, we analysed two different kinds of measurements during the decisions. Firstly, we looked at the choice pattern and secondly RT, to test choice preference and deliberation i.e. what participants paid attention to, how long for, and when. Of key clinical interest here are three facets of the task. First, there is a behavioural measure of decision inertia, i.e. a 'stuck in a rut' bias. Second, it is possible to measure insensitivity to costs during a sequence of searches. Third, it is possible to examine 'pre-emptive avoidance' that occurs at the beginning of the sequence and which is aimed at preventing the bad consequences of such biases when they occur later in the sequence. We will first describe how the behavioural measures are derived from the task. Then we will describe how we have related the cognitive task as well as introspective measures to clinical measures of apathy and compulsivity.

Planning and initiating a search sequence

As previously [9], when deciding whether to accept the default (initial offer) or spin, participants took into account all information that they rationally should (figure S1, results obtained using a decision model using an approach analogous to a logistic regression analysis, see methods 'Analysis – decision'): Participants were more likely to accept the initial offer right away rather than initiate a search, the higher its value (mean=-0.33, 95% CI [-0.33- -0.32]) and the more costly it was to spin the wheel (mean=-0.08, 95% CI [-0.08- -0.07]). In contrast, participants were more likely to search when the average value of the alternatives was high (we refer to this as the myopic value as it only represents the average value of the next search) (mean=0.25, 95% CI [0.25- 0.26]). They were also more likely to search the higher the 'prospective value' (mean=0.05, 95% CI [0.05-0.06]), i.e. the value derived from planning ahead (see supplements section 'Decision tree model' for details on derivation of the prospective value; intuitively, prospective value reflects the additional value that stems from the fact that one can choose to reject bad offers and search again, rather than being stuck with the first offer one gets).

We also found that participants tried, pre-emptively, to avoid situations in which they were subsequently likely to express decision-inertia or insensitivity to cost of searching when performing the sequence of searches (see below, figure S2). To demonstrate this, we first created a measure that captured for each trial how rapidly participants needed to adjust their search strategy as they proceeded through a sequence (AvgProspVChange, i.e. how much the prospective value changes per future search, see methods for details]). The faster the value of searching decreases (i.e. the faster the search strategy needs to be changed), the more expensive later biases are. Thus, if you are aware of your own biases, you should, pre-emptively, particularly avoid starting the search sequence on such trials. This is indeed what we found (mean=-0.03, 95% CI [-0.03 - -0.02]), figure S1A). Relatedly, about half of the participants also self-reported that they pre-emptively avoided trials on which they thought they might over-persevere (figure S1C). We find (see figure 5D, main text) a strong relationship between another sequential bias (cost insensitivity) and self-reported pre-emptive avoidance that is mediated by awareness. Furthermore, our large confirmation sample will give more conclusive evidence about the exact relationship between the many possible biases and their pre-emption in our task, as we could now prespecify the exact relationships to test.

Carrying out a choice sequence

After initiating a search sequence, participants made a series of decisions of either banking an offer (and ending the trial) or searching again (as shown in Figure 2 in main manuscript). We can assess, using the same kind of computational model as above (methods section 'Analysis - decision'), whether participants behave mostly rationally and also whether they have decision inertia, i.e. show a 'stuck in the rut' bias. As before and as they should rationally, we find (figure S2A) that participants are more likely to go on searching if the myopic value is high (mean=0.26, 95% CI [0.26 - 0.26]), if the prospective value is high (mean=0.07, 95% CI [0.07 - 0.07]) or if the cost of searching is low (mean=-0.08, 95% CI [-0.08 - -0.08]) or if the drawn offers are low (mean=-0.49, 95% CI [-0.49 - -0.48]) . We however also

find evidence for decision inertia ('stuck in a rut' bias): the more participants had already searched, the more likely they were to search again (mean=0.04, 95% CI [0.03 - 0.04]). Using our computational model, we could in fact dissociate this from other alternative explanations of searching too often. One possibility might have been that participants only compute the prospective value of a trial once in the beginning of a trial. Then, as they go through as sequence, they would not take into account that as they go through a sequence of searches they must adjust their prospective value because as fewer searches remain there are fewer opportunities to encounter good offer and reject bad ones. However, we find that participants in fact adjust their prospective value ('ProspVS1-Adapted', mean=-0.03, 95% CI [-0.04 - -0.03]). Another possibility might have been – and which is what we assumed previously – that participants have a sunk cost fallacy, i.e. that they are more likely to continue searching the more costs they've already paid on a trial. However this was not the case ('TotalCost' is not positive, (mean=-0.00, 95% CI [-0.00 - -0.00]). In terms of subjective experience, participants reported both 'decision inertia', i.e. searching too often just because they had already searching (about half of the participants, figure S2C) and 'over chasing' of alternatives, i.e. searching too often because an alternative on the wheel appeared particularly appealing, (figure S2D).

RTs, deliberation, and adjustments during search initiation and maintenance
We also analysed participants' RTs. We again split the behaviour into initiation (figure S1B) and the later search sequence period (figure S2B). We found that myopic value only sped up choices in the initial decision (mean=-0.06, 95% CI [-0.07 - -0.05]), but not during the later searches (mean=0.00, 95% CI [-0.00 - 0.01]). Contrary to this, costs, despite also not changing during the course of a given search sequence, had a similar effect on both initial and later RTs, slowing down participants in both cases (mean=0.06, 95% CI [0.06 – 0.07], mean=0.03, 95% CI [0.02 - 0.03]). Prospective value slowed down initial decisions (mean=0.06, 95% CI [0.05 - 0.06]), as it required extended planning to compute. During the later searches, a need to adjust prospective value as fewer opportunities remained, slowed participants down ('ProspS1-Adapted', mean=0.03, 95% CI [0.02 - 0.03]). Additionally, during later searches, every subsequent commitment to search further sped up the next decision, leading to an increasingly speeded response together with increasingly biased choices in favour of search ('# Prev. searches', mean=-0.07, 95% CI [-0.07 - -0.06]).

References

1. Ang Y-S, Lockwood P, Apps MAJ, Muhammed K, Husain M. Distinct Subtypes of Apathy Revealed by the Apathy Motivation Index. Gulinello M, editor. PLOS ONE. 2017;12: e0169938. doi:10.1371/journal.pone.0169938

2. Foa EB, Huppert JD, Leiberg S, Langner R, Kichic R, Hajcak G, et al. The Obsessive-Compulsive Inventory: Development and validation of a short version. Psychol Assess. 2002;14: 485–496. doi:10.1037/1040-3590.14.4.485

3. Beck AT, Steer RA, Brown GK. Beck depression inventory-II. San Antonio. 1996;78: 490–498.

4. Spielberger CD. Manual for the State-Trait Anxiety Inventory STAI (form Y)(" self-evaluation questionnaire"). 1983.

5. Liebowitz MR. Liebowitz social anxiety scale. CDocuments SettingsWindowz XPMy Doc Anxiety Community-Soc Anxiety Disord Liebowitz Soc Anxiety Scale Htm. 1987.

6. Mason O, Linney Y, Claridge G. Short scales for measuring schizotypy. Schizophr Res. 2005;78: 293–296. doi:10.1016/j.schres.2005.06.020

7. Michielsen HJ, De Vries J, Van Heck GL. Psychometric qualities of a brief self-rated fatigue measure. J Psychosom Res. 2003;54: 345–352. doi:10.1016/S0022-3999(02)00392-6

8. Bagby RM, Parker JD, Taylor GJ. The twenty-item Toronto Alexithymia Scale—I. Item selection and cross-validation of the factor structure. J Psychosom Res. 1994;38: 23–32.

9. Kolling N, Scholl J, Chekroud A, Trier HA, Rushworth MFS. Prospection, Perseverance, and Insight in Sequential Behavior. Neuron. 2018;99: 1069-1082.e7. doi:10.1016/j.neuron.2018.08.018
