# Supplementary figures and images for: The effect of apathy and compulsivity on planning and stopping in sequential decision-making
Source: PLoS Biol. 2022 Mar 31;20(3):e3001566. doi: 10.1371/journal.pbio.3001566 (PMC8970514; doi:10.1371/journal.pbio.3001566)

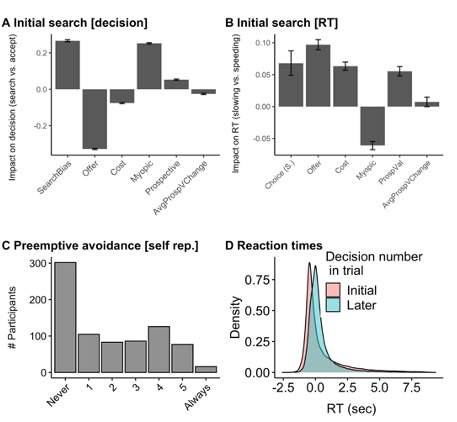

Supplement: S1 Fig — (A) Using a decision model, we tested which factors influenced the decision of whether to initiate a search. We found that participants were more likely to search when prospective and myopic values were high and that vice versa they were less likely to search when initial offer value or costs of searching were high. (B) We analysed the impact of different factors on RTs using a regression analysis. Of particular note, the larger the prospective value, the slower participants responded, potentially indicating more time taken to plan ahead. (C) When directly asked about their preemptive avoidance about half never reported using such strategies (self-report Q3). (E) Histogram of RTs across all participants, for initial searches on each trial (red) and later searches (blue). Note that for initial decisions there was a fixed delay of 3 to 6 seconds on each trial before participants could make responses, which is not included in the RT measured here. RTs for initial searches have an earlier mode than for later searches, but more slow RTs (“thicker tail” of the distribution). Error bars show Bayesian 95% credible intervals (2-tailed), significance is shown by credible intervals not including zero. Data of A in file 11, B in 12, C in 2, and D in 1. RT, reaction time. (TIFF) [file pbio.3001566.s002.tiff]

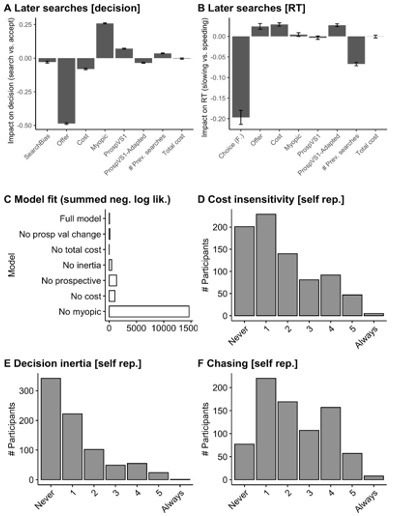

Supplement: S2 Fig — (A) Once participants engaged with a sequence of searches, they had to decide how long to go on for. We found evidence for decision inertia (“# Prev. searches”) that was independent of effects of “sunk cost” fallacy (“Total cost”) or lack of updating of prospective value, i.e., their search strategy (“ProspVS1-Adapated”). Across all participants, we found sensitivity to costs (“Cost”). (B) RTs for later searches revealed that the more often participants had searched already within a given sequence, the faster they got (“#Prev. searches”). Moreover, as in the initial search (S1B Fig), the higher the cost, the slower they responded on these later searches in the sequence (“Cost”). (C) Model fits for the decision data, higher values indicate worse model fit (sum of log likelihoods across all participants and all trials, computed using cross-validation, relative to the best fitting model). The “full model”—the model used throughout the manuscript—provides the best fit to the data. The other models were derived from the “full model,” leaving out individual components of the model, one at a time. (D) Participants were also asked about their own task behaviour, such as their perceived cost insensitivity and most reported insensitivity to the costs to some extent (question only included in the confirmation sample). (E) Despite the strong choice effects of decision inertia, about half of the participants reported having no such bias (self-report Q 1). (F) Contrary to this, about 80% of people reported having been biased towards overchasing a rewarding option at least somewhat (self-report Q2). Error bars show Bayesian 95% credible intervals (2-tailed), significance is shown by credible intervals not including zero. Data of A in file 9, B in 10, C in 15, and D to F in 2. (TIFF) [file pbio.3001566.s003.tiff]

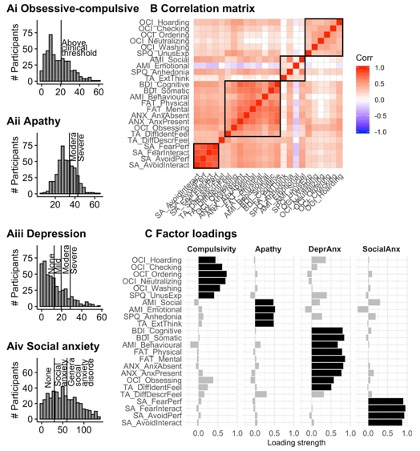

Supplement: S3 Fig — (A) Histograms of the distribution of total questionnaire scores for the OCI-R (Ai), the AMI (Aii), the BDI (Aiii), and the Liebowitz social anxiety scale (Aiv). On each histogram, cutoffs based on previously published normative data are highlighted. (B) Correlation matrix (Pearson’s r) for all subscales included in the factor analysis. Squares highlight the factors subscales were assigned to in the factor analysis. (C) Loadings (i.e., the contribution of each subscale to each factor) of the factor analysis of the subscales. Highlighted in black are loadings above 0.4, for ease of visualisation. AMI, Apathy Motivation Index; BDI, Beck Depression Inventory; OCI-R, Obsessive-Compulsive Inventory. (TIFF) [file pbio.3001566.s004.tiff]

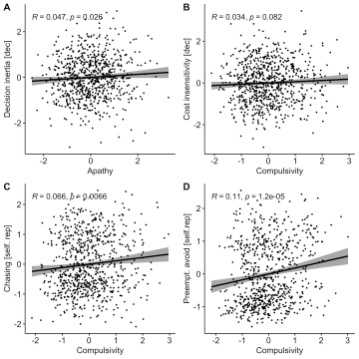

Supplement: S4 Fig — Significance was assessed using nonparametric correlations (Kendall’s tau). Panel A corresponds to regression coefficients shown in Fig 4. Panels B–D correspond to regression coefficients shown in Fig 5 A–C. Note that for correlations of clinical factors with self-report questionnaires (C and D), responses needed to be treated as continuous, while in the regression analysis they were treated as ordered factors. Data of A to D in file 9, 2, and 16. (TIFF) [file pbio.3001566.s005.tiff]

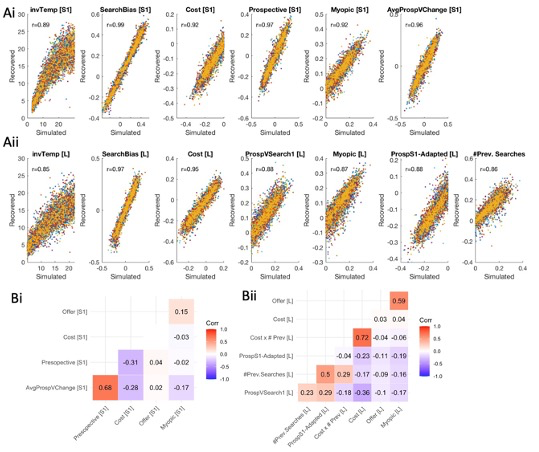

Supplement: S5 Fig — We constructed 10 random schedules as detailed in the S1 Text section “Task schedule design and model validation.” Scatter plots show correlations between simulated and recovered parameters for the initial search (Ai) and later searches (Ai). Different colours show the different schedules (each with n = 500 simulated participants). Correlations (Pearson’s r) are shown across all 10 schedules. The average correlations (Pearson’s r) between regressors used in the RT regression and the decision-making models are at or below 0.7 for all variables for initial decisions (Bi) and later (Bii).” Data of A in file 13 and 14 and B in 1. (TIFF) [file pbio.3001566.s006.tiff]
