# Supplementary material for: The effect of apathy and compulsivity on planning and stopping in sequential decision-making
Source: PLoS Biol. 2022 Mar 31;20(3):e3001566. doi: 10.1371/journal.pbio.3001566 (PMC8970514; doi:10.1371/journal.pbio.3001566)
Supplement: Attachment — Submitted filename: Reviewer_Letter_reducedFileSize.docx [file pbio.3001566.s023.docx]

**REVIEWERS' COMMENTS:

Reviewer #1:

My understanding is that this is a "stage 1" paper that essentially provides context, study design, and pilot data. I review on that basis.

The authors plan to examine decision-making and compulsivity. The topic is very important and interesting. I enjoyed reading the paper and feel it has a number of novel aspects.**

*We thank the reviewer for taking the time to assess our paper and their helpful comments.*

**Comments: 

1. The authors have already conducted a large study (n=449). Please clarify how the planned study is different - if it is simply collecting more data using an identical protocol, and the same recruitment pool (albeit in a new sample), then the presented data here aren't pilot data but a large part of an already substantially advanced study. This is fine but if so I don't think this is a 'stage 1' paper in the sense I have understood it (my understanding is that stage 1 is essentially "pre-registration" of methods for a study you plan to do in advance of doing the study). Put differently, if the study is already quite advanced in progress, shouldn't all the data just be reported in one paper, as it isn't "pre-registration"?**

*Firstly, we apologize for not being clearer about the rationale behind our procedure. In short, our goal was to use data-driven methods to refine our prior hypotheses using the discovery sample and test those in our pre-registered confirmation sample. What we are effectively pre-registering in this study is the statistical tests we are going to do based on the discovery sample we have collected.*

*Specifically, we converged on the following two step process: first we have collected a ‘discovery sample’ (n=449). This is the data presented here that forms the basis of this registered report stage 1 submission. Based on this sample, we performed power calculations to determine the required sample size to replicate the effects (n=750, with power=0.95). The second sample will be analysed using exactly the methods described here. Importantly, however, it will be analysed separately from the first sample, rather than combining the two samples.*

*Based on the reviewers comment we made our rationale clearer in the current document in the following way:*

Introduction [as a new last paragraph]

For data collection and analysis we employed a ‘split sample’ approach [33,34]. We first collected a discovery sample which we used to refine and develop hypotheses in a data-driven way. We then used a confirmation sample to test the pre-registered hypotheses in a statistically rigorous way. This approach thus combines the benefits of pre-registration - reduced risk of false positives due to analytical freedoms - with those of exploratory science – the analytical approach being best adapted to the specific data.

**2.  "We collected questionnaires with measures covering a wide range of psychiatric symptoms....."

Only one type of compulsivity is considered in the study design (OCD) as far as I can discern. I appreciate some papers in the literature uses OCD symptom scales (e.g. OCI; Padua...) as a putative 'trans-diagnostic' measure but it isn't really, as all the items are specifically to do with OCD and not the vast range of other types of compulsive symptoms (including those mentioned by the authors). I would encourage the authors to incorporate a broader range of symptoms into their 'compulsivity' measure. Or if they focus on OCD, this is fine, but actually it should be referred to as "OCD-related compulsivity", rather than a trans-diagnostic measure.**

*We agree with the reviewer and have now clarified the type of the compulsivity we look at using the questionnaires as “OCD-related compulsivity*” in the main text.

Introduction:

OCD-related compulsivity (measured here using questions from OCD and schizotypy inventories; ‘compulsivity’ for short) will be linked to…

Discussion:

**Compulsivity**

The dimension we called ‘compulsivity’ included different subscales of the obsessive compulsive inventory, with the notable exception of the obsessive thinking subscale, as well as the unusual experience subscale of the schizotypy questionnaire. We did not include broader questions measuring compulsivity in the context of other disorders such as eating disorders or gambling.

**3. More detail should be provided about how the participants (and future participants) were recruited and selected. People doing online platforms may be unrepresentative of the general population. Also give thought to: are data collected online clinically valid? Is the cognitive task valid administered over the Internet? This may be the case but please provide more / clearer information. It would be important to list clearly as a limitation that online data collection is not the gold standard and may not generalise to findings collected in-person using trained raters, objective measures, and supervised cognitive tests.**

*We thank the reviewer for raising these points and agree that it is very important to characterize one’s sample to relate it to the general population.*

*We, and others* [3–5], *have found that in terms of psychiatric scores, participants online span a wide range, from normal to clinically relevant (figure 2A in manuscript, shown again below). Particularly some symptoms, like social anxiety appear to be more prevalent online than in the general population. We have now included an additional table showing the distribution of demographic variables in our sample. This highlights that our sample is well-balanced in terms of gender and relatively young (inclusion criteria were 18 to 40 years).*

Table R1 (Table 1 in main text now): Demographics and duration of experiment for participants that we included or excluded from all analyses based on questionnaire and task performance criteria (see Methods for details).

*Concerning the clinical validity of the questionnaire scores collected over the internet, our study cannot address this directly as we have no ground truth diagnostic information. However, a very recent study from Gillan et al. [6] set out to answer exactly this question. They found that responses online and in an in person interview are comparable. Crucially, this study also highlighted that in fact dimensions of e.g. compulsivity – as we also derive here – can even be more robustly related to behavioural test measures than diagnostic labels.*

*Concerning the validity of the task over the internet, we took several steps to ensure high data quality: First, we ensured participants fully understood the task instruction by giving them an automated tutorial followed by a multiple choice test that they needed to pass with 100% correct before being able to do the task. Second, we gave participants bonus payments depending on their performance to increase motivation. Third, we checked participants’ behaviour for sufficient task understanding (see exclusion criteria); in fact, this check revealed that only 2 out of over 450 participants who had passed the multiple choice test were not able to do the task. Fourth, we analysed the behaviour of participants online with the same analysis pipeline as described previously [ref Kolling & Scholl, 2018], which revealed very similar patterns of behaviour in the lab and online. Thus, we think that our on-line behavioural task data are similar to cognitive task data obtained under more controlled conditions in the lab. We realise, however, that this could be explained more clearly and so we have attempted to do this in the revised manuscript (discussion).*

In introduction:

[…] This is particularly relevant for three reasons. First, many disorders, such as obsessive-compulsive disorder (OCD), have co-morbidity rates as high as 90% [7]. Secondly, it is well known that some dimensions, are shared across many disorders [8]. Third, recent work [6] suggests that dimensions cutting across disorders relate more strongly to behavioural deficits than diagnostic categories.

Discussion [section ‘transdiagnostic approach and ecological paradigm’]

Teasing apart the role of distinct yet partially correlated transdiagnostic symptoms was made possible here by testing a large number of participants over the internet. For this approach to work well, it is necessary that symptom scores span the range from healthy to psychiatrically relevant. We, as others previously [36,48,49], indeed found here that participants in an internet based study showed this range of symptoms and in fact even showed symptoms at higher rates than found in the normal population. To ensure that the self-reported symptoms were valid, we included several quality checks. We also note that previous work [20] that directly compared transdiagnostic dimensions derived from self-reports over the internet to clinician rated diagnoses found good correspondence between them and crucially also that dimensions related more clearly to behaviour than diagnostic labels.

[…] We complemented this approach with analysis of participants’ self-reports of their own behaviour to test their meta-cognition. We ensured good data quality by giving participants automated training on the task and testing their knowledge using a multiple choice test they had to pass before being admitted to the main study. As a consequence, data quality was very high (only two participants had to be excluded based on task performance).

In Methods [section ‘participants exclusion criteria’]:

As our data were acquired online, we took several steps to ensure data quality. First, we ensured participants fully understood the task instruction by giving them an automated tutorial followed by a multiple choice test that they needed to pass with 100% correct before being able to do the task. If they failed the test, they could retake the tutorial and the test. Second, we gave participants bonus payments depending on their performance to increase motivation. Third, we excluded participants based on task performance as well as poor completion of the questionnaires.

**4. Consider whether exclusion based on questionnaire responses, as listed, might be too strict. You will probably knockout people in your sample who are particularly compulsive and/or have attentional abnormalities (common in the setting of compulsivity). Best would be to undertake analyses with full datasets and compare findings using these more rigorous criteria. That way you address the issue but don't have to worry about biasing your sample.**

*The reviewer is making an interesting point. We worry that in our discovery sample some participants might have filled in the questionnaire inappropriately due to many possible reasons that could contaminate the results beyond their symptoms of compulsivity. To look at this further, we refer to the table R1 above that shows measures of durations of the different experiment components for participants who were either included in the study, excluded because of insufficient trials for specific behavioural analyses or based on our questionnaire criteria. We noted that participants excluded based on questionnaire measures took significantly longer to complete the training or the main task. As on the internet platform there was a ‘time out’, this might have made participants try to complete the questionnaires very quickly to finish in time. We will address this in the confirmation sample by increasing the permitted time for the whole study, as well as giving participants warnings if they complete questionnaires quickly (faster than reading time).*

*We hope that this will mean that fewer participants will need to be excluded based on questionnaire completion measures. In this ‘cleaner’ sample we will then be able to compare behavioural measures of the excluded and included participants.*

*We have now also further simplified our list of exclusion criteria based on questionnaires* :

Exclusion based on questionnaire responses:

1) Not completing all psychiatric questionnaires

2) Five questions will be repeated. If a participant’s responses are different by two or more points on average, they will be excluded.

**5. Statistical approach is very convoluted for the decision-making task and its outcome measures. What will these measures mean for clinicians and patients? How easy will it be for other groups to replicate any results, unless they have a PhD in statistics? How many complex statistical assumptions are made that may or may not be valid?  This is the reviewer's clarion call to the authors for well-validated straight forward decision-making task with clear easy-to-understand outcome measures. The reviewer appreciates he appears to be fighting a losing battle in the field of decision-making in this regard. If it really is necessary to use such a complex approach, please explain why, and provide an overview of the steps in language that a typical person reading a psychiatric or neuroscience journal would understand; aside from also providing the very detailed information too.**

*We agree with the reviewer in retrospect that we could have done a better job at making our rationale clearer.*

*In essence, in the paper we combine two different types of models. The first model is a ‘decision tree search model’. We use this model to determine the ‘search value’ for each trial. The search value was made up of two components: the myopic value was simply the weighted average of the alternatives in the environment. While the ‘prospective value’ captures the value of this environment beyond the average, which is there because participants can search repeatedly. We agree with the reviewer that this model might not be intuitive to the reader. To address this, we have now included a new figure that illustrates what this prospective value is based on (figure R2 below). We find basically, and in agreement with intuition, that the more often participants can search and the more variable the environment is (higher standard deviation), and the lower the cost of searching, the higher the value of the environment for an individual that plans ahead. To give an intuitive example, if there is one very valuable offer that is at the same time very unlikely (i.e. only has a small area on the wheel), then it matters whether I can search many times (and how expensive it is to search. If I can search for it again and again then, eventually, I will land on that offer. If, however, I cannot search repeatedly, or if it is too costly to search repeatedly then I may well never land on that on option. In such cases the long term prospective value of the environment is lower. Importantly, all features of the scenario presented on each trial interact with one another (is there a high value option out there, what are the chances of landing on it, how many opportunities to do so do I have, how costly is it to take each opportunity) and the key thing about the model is that it sets out a quantitative explanation of how the different aspects can be integrated. In other words, the cognitive construct of prospective value is built upon all these model elements.*

Figure R2 (included in figure 1D). Prospective value is derived from a decision tree model (see supplementary methods). This model captures the way in which prospective value is higher in environments with many available searches (num. searches), higher variability in how good and likely the offers are (standard deviation, STD), particularly if many searches are available (STD*Num. searches) and low cost, again particularly if many searches are available. Results are regression weights relating prospective value to different features of the environment (see Methods). The proportion of variance of prospective value explained by the regressors (R^2^) was 80%.

*The second kind of model we used was a standard multiple regression model. We used this model to measure how specific features of each trial affected participants behaviour. The two features that we were particularly interested in regarding clinical features were the cost of searching, as well how often participants had already searched on a given trial. We have now added a new figure (R3, below) to help the reader get an intuition for how these features affect behaviour and what our model is trying to capture. In short, the higher the cost of searching (colour), the less likely participants are to search. The individual variation in this factor ('cost insensitivity') is what we relate to compulsivity. Secondly, the more often participants have already searched in a trial, the more likely they are to search again. The individual variation in this measure is what we related to apathy (‘decision inertia’). However, we also note that one downside of using these simple measures is that they do not allow us to consider multiple decision factors in parallel and are in fact partially confounded if multiple factors correlate to an extent. For example, in the figure below, as the number of previous searches increases, so does the search value (as participants are more likely to search frequently in trials with higher search value). The best solution to this problem is multiple regression analysis because it can control for multiple partially covarying factors. We hope that by including the additional figure that shows what features of the raw data the computational model is aiming to capture, we will help to make a link between the data and the model clearer to the reader.*

*In terms of checking whether the assumptions that our models make are reasonable, we can – as also suggested by another reviewer – check whether the model captures our data well using posterior predictive checks. This means comparing the behaviour predicted from our model with the behaviour of real participants. If it is similar, it suggests that our model (and its assumptions) are appropriate. To check this, we have now included the model predictions in figure XX below. We find that indeed our model captures our data very well.*

Figure R3 (updated figure 1, panels E+F): Participants’ behaviour together with model predictions. D) The higher the relative value favouring searching vs. accepting the offer, the more likely participants were to search (dots with error bars). Similarly for the model (line). E) Illustration of the main behavioural measures used to relate clinical factors to (figures 3-4). In general, the more often participants (dots with error bars; continuous lines show model predictions) have already searched on a given trial, the more likely they were to search again, rather than accept an offer (‘decision inertia’). Note that in contrast to the regression models (figure S1-S2), this split does not correct for the fact that other factors such as search value are partially correlated with the number of previous searches but it does provide a simple way to visualize the impact of task features on participant behaviour. The graph also shows the higher the cost (grey to blue), the less likely participants were to search. Error bars show the standard error of the mean, size of the dots show the mean number of data points per participant and lines show the model predictions.

*Finally, in terms of the analysis being useable by other groups, we will make all scripts available online on publication in a format that is well annotated, and which allows for easy running of analyses.*

*Methods [section ‘ Analysis -decision’] :*

We checked how well the behaviour of the fitted model resembled the behaviour of real participants (posterior predictive checks). For this, we extracted, for each participant and for each search decision, the model’s probability of searching as opposed to accepting the offer, given the parameters of the best fitting model. These are shown in figure 1E-F together with the data from real participants.

Methods [section ‘computational model of prospective value’]:

*We illustrate the key factors contributing to our model-based prospective value estimate (see figure 1D). For this we first conducted a regression predicting prospective value based on the average value of all alternative options in the environment (myopic value), the standard deviation of all alternatives, the number of searches available, the cost of searching, and all possible two- to four- way interactions. To compute how well these components together approach prospective value, we computed the variance explained (R^2^).*

Results [section ‘Validation of sequential decision task’]:

Validation of the paradigm in terms of general findings can be found in the supplements (supplementary results and supplementary figures S1-S2); the model captured participants’ behaviour well (figure 1 E-F).

**6. CFA will be done in independent data-set; correct?**

Yes. Confirmatory factor analysis will be performed on the replication data set to test that the factor solution obtained from the exploratory sample replicates.

**7. Have all confounds be considered - what about IQ, years of education, psychoactive medications, etc.?**

*In the discovery sample, we controlled for age, gender and years of education. Based on the reviewers’ comments we also added a question on participants’ prescribed medication (specifically, we will ask participants to indicate what they are currently taking and will then code this based on the mechanism of action, e.g. serotonergic, noradrenergic drug). While we cannot add a full IQ test because of time constraints, we do control for years of education.*

Methods [section ‘Questionnaires’]:

To obtain additional control covariates for analyses, we also asked participants about their age, gender, years of education (GCSE, A-levels/High school, BSc, MSc, PhD) and whether they were currently taking any psychoactive medication or drugs (which will be coded as distinct categories whenever drugs listed by at least 10 participants fell in a category, e.g.: serotonergic, noradrenergic, dopaminergic, GABA-ergic and ‘other’; otherwise just listed as ‘psychoactive medication yes/no’) and, if applicable, frequency and dose.

**Reviewer #2:
[identifies himself as Jiaxiang Zhang]**

**[The importance of the research question(s)]**

**The current pre-reg report aims to collect behavioural responses from a large online sample (>650). The sequential decision-making task to be used is from the authors' previous study, which ensures feasibility of the proposed study and validity of data collection. The large online sample enables addressing new important questions, specifically how apathy and compulsivity traits may relate to differences in choice behaviour across participants.**

**[The logic, rationale, and plausibility of the proposed hypotheses]
The ms provided a good rationale for the proposed research, including the need for using transdiagnostic trait measures. Three main hypotheses have been identified, relating (1) apathy to decision inertia, (2) compulsivity to cost insensitivity and (3) compulsivity to self-report measures.**

**I hope the authors could clarify the following two issues:**

**1. The ms referred to clinical populations (OCD and depression) when justifying the hypotheses. Can the current and proposed results rule out an alternative explanation that the trait-performance linear association is mainly/disproportionally contributed by those individuals with more extreme trait measures (as in Fig. 2A)?**

*We thank the reviewer for taking the time to read our paper and their thoughtful comments.*

*To answer their first question about what drives the correlations we observed, we now plot the correlations, including non-parametric statistical tests that are less influenced by outliers. The figure shows that the correlation is not driven primarily by a strong association around the extremes of the measurements.*

Figure R4 (new figure S6). Partial correlations between clinical factors and behavioural measures, controlling for other clinical, demographic and general task performance measures (see methods ‘Relating questionnaires to task performance and self-reports’. Significance was assessed using non-parametric correlations (Kendall’s tau). Panels A-B correspond to regression coefficients shown figure 3A-B. Panels C-F correspond to regression coefficients shown in figure 4 A-D. Note that for correlations of clinical factors with self-report questionnaires (E and F), responses needed to be treated as continuous, while in the regression analysis they were treated as ordered factors.

*Methods [Section ‘Relating questionnaires to task performance and self-reports’]:*

To illustrate the relationship between psychiatric factors and behaviour, we also plot partial correlations of the residuals. Residuals were computed in two regressions. First the behaviour of interest was predicted as a function of all factors (i.e. psychological and control measures) listed above, apart from the one of interest (e.g. apathy). Second, the residual variance in the behaviour was correlated with the questionnaire dimension of (e.g. apathy) using non-parametric correlations (Kendall’s tau) to ensure results were not driven by outliers. For the analyses of the self-report questionnaire scores, residuals could only be computed by treating them as continuous.

**2. The link between high apathy and excessive effort is indeed counterintuitive. I wonder why, in the current sequential decision task, participants with high apathy trait did not exhibit any avolitional types of behaviour, e.g., longer RT in the first decision (initial search) of each trial?**

This is indeed an interesting question. We have tried to give an explanation for the speeding within the sequence, but fear that our task was not ideally set up for examining reaction time in the initial decision, because we enforced a relatively long monitoring/wait period (3 to 6 seconds) before participants could indicate their first choice. This was to ensure participants would always observe all the options and information, at least initially. This could have affected any effects of avolition on fast responding that we might have observed otherwise. However, given that participants are encouraged to at least initially assess their environment, it is worth noting that we do find that participants with apathy show decision avolition (don’t easily change the kind of decision they make from search to accept) instead of action avolition (give up prematurely). While future studies might examine reaction time more effectively with very different experimental tasks from the one we use here, we think that we can demonstrate a quite compelling decision avolition effect. We now try to make this clearer in the manuscript:

Introduction:

*We can also measure how participants can fail to adjust through two biases: first, participants’ can be insensitive to repeatedly incurred costs of decisions as they progress through a sequence. Second, participants can have decision inertia, i.e. inflexibility and repetitiveness. This is distinct from action inertia, which would lead to increased reaction times and no corresponding change in choices.*

Discussion [section ‘Apathy’, second paragraph]:

We also note that we did not find an effect of apathy on general response slowing at the initial decision. This was potentially so because our task had a fixed ‘monitoring’ period (3-6 sec) on each trial to ensure participants considered all information before responding, but potentially reducing sensitivity to individual differences in RT.

Discussion [last paragraph on apathy]

[…] as well as experimental findings of reduced generation of new options as candidates for decision making. We note that this finding of decision inertia might be distinct from a general response slowing, which we did not observe. However, we also note that before the initial choice of each trial, there was a forced delay of three to seven seconds to ensure participants considered all options and attributes. This could have reduced the sensitivity of this analysis to the effects of apathy.

**[ The soundness and feasibility of the methodology and analysis pipeline ]
The authors conducted rigorous analysis on an existing online dataset, with detailed description of methods and power analysis for the proposed study. The protocol (paradigm, hierarchical modelling and analyses) is technically sound and appropriate for testing the stated hypotheses.**

**The author should provide a more explicit list on all the differences in data/analysis between the discovery (existing) and confirmation samples, as they scattered across the current ms (one-tailed/two-tailed, changes in questionnaires, changes in schedules, etc.).**

We thank the reviewer for this valuable suggestion. The table below is now included.

*Table R2 (new table 4 in manuscript). Differences between the discovery and confirmation sample.*

**[ Whether the clarity and degree of methodological detail is sufficient to exactly replicate the proposed experimental procedures and analysis pipeline.]
I am confused why "… 10 random task schedules …" were constructed. Will a subset of participant be tested with an identical task sequence, or will each participants be presented with an unique schedule?**

*We constructed 10 random task schedules, each tested for good parameter recovery (supplement figure S7 [S6 before]). This means that any given participant will share the schedule they performed with a tenth of the other participants. The reason for including 10 schedules was to strike a balance between the risk of spurious results due to an idiosyncrasy of a single task schedule and the risk of making it harder to compare the behaviour of participants who completed different schedules. However, figure S6 (see again below) suggested that parameter recovery was likely to be good within and between schedules.*

**Figure S7. Parameter recovery**. We constructed 10 random schedules as detailed in the supplementary methods section ‘Task schedule design and model validation’. Scatter plots show correlations between simulated and recovered parameters for the initial search (top) and later searches (bottom). Different colors show the different schedules (each with n=500 simulated participants). Correlations (Pearson’s r) are shown across all 10 schedules.

Methods [section ‘Task’]:

Design of task schedule: We constructed 10 random task schedules that fulfilled the following criteria (see Supplementary Methods, section ‘Task schedule design and model validation’ and figure S6 for details): First, correlations between variables influencing decisions (see analyses below for details) were below 0.7. Second, parameter recovery showed Pearson correlations of r>0.7 between simulated and recovered parameters for all parameters. Third, parameter recovery across all schedules showed r>0.7 for all parameters. Any given participant will share the schedule they performed with a tenth of the other participants**.**

**[ Whether the authors have pre-specified sufficient outcome-neutral tests for ensuring that the results obtained are able to test the stated hypotheses, including positive controls and quality checks]
Yes

[Minor]
P25. "Participants were did not adapt …"**

Apologies for the typo. We have now corrected it.

**Reviewer #3:
[identifies himself as Christoph W. Korn]

Scholl et al. propose an exciting study for a registered report: In a large online study, they use a validated sequential risky decision-making task in conjunction with a computational model to dissociate effects of apathy and compulsions and then link these to individual differences based on a transdiagnostic clustering of various questionnaires (pilot sample n>440; proposed sample n=650). I and a PhD student who helped me compile this review are generally in favor of seeing this study conducted. Here is a short assessment of the criteria for Stage 1 enlisted in the reviewer instructions (followed by more detailed specific points):
a)      The research questions are important from a theoretical and an applied view (e.g., theoretical: how well can constructs such as apathy and compulsivity as derived from questionnaires be linked to features from computational analyses of decision-making tasks?; applied: how could this inform research on psychiatric spectrum conditions?).
b)      The logic of the hypotheses is rather valid and clear. The authors could make it a bit easier for the readers to understand the modelling approach (see comments below).
c)      The study is definitively sound and feasible. The current data and results are already quite substantial and the overall analyses are appropriate and sophisticated.
d)      The methodological descriptions are detailed and allow for an exact replication.
e)      The authors have conducted quality tests for their pilot data and one can assume that they will do the same for the main study. Some more quality checks are proposed below.**

**Specific points:**

**1)      Registered report:
a.      Motivation for registered report: The authors do not clarify why they want to conduct a registered report (rather than publishing their current data as they are). Of course, there are numerous advantages to a registered report. We feel that the specific reasons should be stated briefly.**

*We would like to thank the reviewers for their thorough review and helpful comments. Turning, to the specific question of the motivation for a registered report, we apologize for not being clearer about the rationale behind our procedure. In short, our goal was to use data-driven methods to refine our prior hypotheses using the discovery sample and test those in our pre-registered confirmation sample. What we are effectively pre-registering in this study is the statistical tests we are going to do based on the discovery sample we have collected.*

*Specifically, we converged on the following two step process: first we have collected a ‘discovery sample’ (n=449). This is the data presented here that forms the basis of this registered report stage 1 submission. Based on this sample, we performed power calculations to determine the required sample size to replicate the effects (n=750, with power=0.95). The second sample will be analysed using exactly the methods described here. Importantly, however, it will be analysed separately from the first sample, rather than combining the two samples.*

*Based on the reviewers comment we made our rationale clearer in the current document in the following way:*

Introduction [as a new last paragraph]

For data collection and analysis we employed a ‘split sample’ approach [33,34]. We first collected a discovery sample which we used to refine and develop hypotheses in a data-driven way. We then used a confirmation sample to test the pre-registered hypotheses in a statistically rigorous way. This approach thus combines the benefits of pre-registration - reduced risk of false positives due to analytical freedoms - with those of exploratory science – the analytical approach being best adapted to the specific data.

**b.      Approach for registered analyses: Overall the methods are quite clearly described for the current data. But is unclear to us if the authors want to change anything for the new data collection and analyses (except for some changes mentioned in the supplementary file, e.g., changing the number of questionnaires). Put differently, have we learned anything from the current data that would suggest potential improvements for the new study? If not, that's fine and it would help the readers to know what exactly changed between the first and second study, even nothing changed.**

*We thank the reviewers for this suggestion and have now included a new table that lists all changes between the discovery and confirmation sample. The main changes based on the discovery sample were: 1) reduction of the number of questionnaires and number of trials to reduce the experiment duration, 2) improved instructions about importance of questionnaires for the study and checks on participants completion times while completing the questionnaires, 3) simplification of exclusion criteria for questionnaires by including ‘repeated questions’ to check responding.*

**Table R5 (Table 4 in revised manuscript).** Differences between the discovery and confirmation sample.

**2)      Computational model and hypotheses:**

**a.      Description in introduction and main text: It seems to us that the authors want to spare readers the technical details of the model in the main text. This comes at the cost of a rather blurry understanding of how the hypotheses are tested. For example, the second paragraph of the introduction lists what the model can achieve but this remains rather fanciful without reading the methods, which makes the paper's methods appear less robust than they actually are. Some more technical details would help (similar to the very helpful Table 2). In particular, the authors should state clearly in the main text how they can dissociate H1 and H2 since both are superficially linked to "searching longer for a better option." Even readers not versed in modelling could understand that H1 relates more to the number of previous searches while H2 relates to the search costs, which vary across trials. Also, it should be made clear that H3 is based on asking participants a separate
(meta-cognitive) question about the task.**

*We thank the reviewers for pointed this out. We indeed tried to avoid getting into the technical details early on, but as the reviewer points out, perhaps became a bit too vague as a consequence. We now have changed the text in the main manuscript, particularly in the Introduction to make the specifics clearer (see below).*

*Introduction:*

We can also measure how participants can fail to adjust through two biases: first, participants’ can be insensitive to repeatedly incurred costs of decisions as they progress through a sequence. Second, participants can have decision inertia, i.e. inflexibility and repetitiveness. This is distinct from action inertia, which would lead to increased reaction times but no change in which decisions are taken. Figure 1F illustrates both of these biases. Sensitivity to costs is the degree to which searching is made less likely by increasing costs. Decision inertia is the likelihood of deciding to search yet again because you have searched already; when there is decision inertia then the higher the previous numbers of searches then the more likely you should be to search yet again. Formally, we can use our computational models to get quantitative measures of each of these biases, while controlling for all other factors that might affect choices. Finally, we can also measure how participants’ knowledge of their own biases, enables them to pre-emptively avoid situations that are more likely to trigger a bias. We measure this here by asking participants to self-report whether they pre-emptively avoided these situations.

*Additionally, we also followed the reviewers’ suggestion of showing a model-free plot that shows how cost and previous number of searches should affect choices (figure R5, below). For comments on the posterior predictive checks also shown in this figure, please see answer to relevant reviewer question below.*

Figure R5 (updated figure 1, panels E+F): Participants’ behaviour together with model predictions. D) The higher the relative value favouring searching vs. accepting the offer, the more likely participants were to search (dots with error bars). Similarly for the model (line). E) Illustration of the main behavioural measures used to relate clinical factors to (figures 3-4). In general, the more often participants (dots with error bars; continuous lines show model predictions) have already searched on a given trial, the more likely they were to search again, rather than accept an offer (‘decision inertia’). Note that in contrast to the regression models (figure S1-S2), this split does not correct for the fact that other factors such as search value are partially correlated with the number of previous searches but it does provide a simple way to visualize the impact of task features on participant behaviour. The graph also shows the higher the cost (grey to blue), the less likely participants were to search. Error bars show the standard error of the mean, size of the dots show the mean number of data points per participant and lines show the model predictions.

*Methods [section ‘ Analysis -decision’] :*

We checked how well the behaviour of the fitted model resembled the behaviour of real participants (posterior predictive checks). For this, we extracted, for each participant and for each search decision, the model’s probability of searching as opposed to accepting the offer, given the parameters of the best fitting model. These are shown in figure 1E-F together with the data from real participants.

Methods [section ‘computational model of prospective value’]:

We illustrate the key factors contributing to our model-based prospective value estimate (see figure 1D). For this we first conducted a regression predicting prospective value based on the average value of all alternative options in the environment (myopic value), the standard deviation of all alternatives, the number of searches available, the cost of searching, and all possible two- to four- way interactions. To compute how well these components together approach prospective value, we computed the variance explained (R^2^).

Results [section ‘Validation of sequential decision task’]:

Validation of the paradigm in terms of general findings can be found in the supplements (supplementary results and supplementary figures S1-S2); the model captured participants’ behaviour well (figure 1 E-F).

**b.      Alternative models: Do the authors test any potentially simpler or more complex models (irrespective of the link to individual differences)? It seems to us that the authors should first show that a given model component is relevant for the whole group of participants before going into questions of individual differences.**

*We agree that including model comparisons is a very standard approach in any modelling paper. The reason we did not do a model comparison is that finding the best model on the group level is a bit in tension with an individual differences approach: a model that is the best at the group level, may exclude factors that have a lot of individual differences, potentially correlating with individual differences in questionnaire self-reports. This is why we focused on using a model we had already established as having high explanatory power in our previous paper, only including additional regressors as confound variables to show the specificity of the reported associations (e.g. sunk cost fallacy vs decision inertia).*

*However, based on the reviewers’ comments, we have now considered several ways of comparing models: First, as included previously in the Supplements (S2, shown again below), we can check whether the included regressors affect behaviour significantly in the group of participants as a whole. Second, we can compare the model fit across participants using a summary statistic under the assumption that each participant uses the same cognitive model. This is in alignment with the rest of our modelling approach of using hierarchical models that incorporate a group level hierarchy (i.e. regression weights of individual participants are normally distributed). For a hierarchical model, computing model fit adjusting for the number of parameters (as e.g. one would compute BIC for a non-hierarchical model) is not trivial as the number of free parameters is not known (it is known that the number will be between the number of group level parameters and the number of individual subject parameters). To overcome this, we chose half-split cross validation. That is, we fitted the hierarchical model on half of the data for all participants and then computed model fit (as log likelihood) on the left-out test-data (repeat process then switching test and training data set). This does then not need further adjustment for the number of parameters included. The results of this are shown in updated figure S2, see below (figure R6). This suggests that there is a benefit for the model that we originally used in the paper.*

*In summary, different modelling indices suggest that the full model which we also used to analyse behaviour throughout the paper provides a good fit to behaviour. We also note that one risk of running model comparisons to identify which model provides the best fit on the whole group is that as a result parameter with large individual differences (i.e. some participants take the parameter into account, others don’t) may be excluded even though these might show meaningful differences with psychiatric dimensions. For this reason, we would err on the side of caution, including a parameter rather than excluding it if the evidence for or against inclusion is not very strong either way. In this vain, the total cost was specifically included here to give us a measure of ‘sunk cost’ which is an alternative hypothesis for why participants might be searching for too long. In other words, we are not invested in whether total costs affect behaviour in the group as a whole or not, but simply wanted to show that any such effects, if they exist, are not enough to explain away our other findings. Thus, we decided to keep using this model throughout as it allows ruling out the alternative hypothesis. However, we also note that when we repeat analyses shown in the main text with the model without the total cost, all results are exactly the same.*

**Figure R6 (updated Figure S2 in supplements). Effects on decisions and deliberation time during later decision sequence.** A) Once participants engaged with a sequence of searches, they had to decide how long to go on for. We found evidence for decision inertia (‘# Prev. searches’) that was independent of effects of ‘sunk cost’ fallacy (‘Total cost’) or lack of updating of prospective value, i.e. their search strategy (‘ProspVS1-Adapated’). Across all participants, we found sensitivity to costs (‘Cost’). B) RTs for later searches revealed that the more often participants had searched already within a given sequence, the faster they got (‘#Prev. searches’). Moreover, as in the initial search (figure S1B), the higher the cost, the slower they responded on these later searches in the sequence (‘Cost’). C) Model fits for the decision data, higher values indicate worse model fit (sum of log likelihoods across all participants and all trials, computed using cross-validation, relative to the best fitting model). The ‘full model’ – the model used throughout the manuscript - provides the best fit to the data. The other models were derived from the ‘full model’, leaving out individual components of the model, one at a time. D) Despite the strong choice and RT effects of decision inertia, about half of the participants reported having no such bias (self-report Q 1). E) Contrary to this, about 80% of people reported having been biased towards over-chasing a rewarding option at least somewhat (self-report Q2). Error bars show Bayesian 95% credible intervals (two-tailed), significance is shown by credible intervals not including zero.

Supplementary methods [section in ‘Task schedule design and model validation’]:

To check how well our decision model fitted our data, we computed the negative log likelihood for each measurement from each participant using a half-split cross-validation procedure. Specifically, for each participant we selected a random half of their data points and then fitted hierarchical models to these ‘training data’. We then applied the parameters to the ‘test data’ (remaining half of data) and computed for each data point the fit (negative log likelihood). As a cross-validation procedure was used, no further adjustment for the number of model parameters was needed. We compared models that contained all regressors of interest, as well as control regressors (see figure S2A), with models where iteratively one of the regressors was removed (figure S2C). The aim was to test how important each of the regressors was for capturing participants’ behaviour. We compared the model fit by computing the sum of the negative log likelihoods across participants, lower values suggest better fit.

**c.      Split of behavior in 1st decision to search and consecutive decisions: Could the authors give a better rationale for this in the main text. Are the RTs of the 1st decision meaningful given that there was a forced (and jittered) delay?**

*We split the data into later and initial decision because we think we cannot assume that decisions are made in the same way at both times. First, the initial decision had a forced delay, meaning that participants were forced to spend time considering their choices. In contrast, later choices could be made immediately. Second, considering the patterns of reaction times, suggests that it may reflect different processes. For example (comparing figure S1B and S2B – S2 shown above and S1 shown again below) shows that at the initial choice, participants are slower to respond if they choose to search, while in contrast, for later searches, participants respond faster if they choose to search; similarly, at the initial search, higher myopic value speeds up participants’ responses, while for later searches it does not impact on RT. We can also compare histograms of participants; RTs for the initial vs. later searches (below, new panel S1D). Qualitatively, we find that the mode of RTs for initial searches (minus the forced delay) are faster than for later searches, but there are more RTs for initial searches at high RTs (‘thicker tail’). Third, importantly there are normative/cognitive reasons to believe that the initial planning decision is different from the later decisions because no new information about the environment is given and later decisions can be pre-planned. In fact, we have some evidence that there is neural activity that looks like pre-planning from our previous paper ([9], figure 6).*

*Finally, we address the question whether RTs for the initial decision are meaningful in any sense. We find that different factors affect RTs and that in several cases (e.g. offer value and cost) the pattern of effects is very similar across initial and later decisions. This suggests to us that despite the forced and jittered delay, RTs still reflect the initial decision process. Of course it is possible that without the forced delay, effects would be even stronger but the fact that they exist is demonstrable with the current paradigm.*

**Figure R7 (updated Figure S1 in supplements). Effects on decisions and deliberation time during initial decisions.** A) Using a decision model, we tested which factors influenced the decision of whether to initiate a search. We found that participants were more likely to search when prospective and myopic values were high. And that vice versa they were less likely to search when initial offer value or costs of searching were high. B) We analysed the impact of different factors on RTs using a regression analysis. Of particular note, the larger the prospective value, the slower participants responded, potentially indicating more time taken to plan ahead. C) When directly asked about their pre-emptive avoidance about half never reported using such strategies (self-report Q3). E) Histogram of RTs across all participants, for initial searches on each trial (red) and later searches (blue). Note that for initial decisions there was a fixed delay of 3-6 sec on each trial before participants could make responses, which is not included in the RT measured here. RTs for initial searches have an earlier mode than for later searches, but more slow RTs (‘thicker tail’ of the distribution). Error bars show Bayesian 95% credible intervals (two-tailed), significance is shown by credible intervals not including zero.

*We now make our rationale clearer in the text:*

*Methods [section ‘analysis – decision’]:*

We analysed the initial search decision on each trial and the subsequent searches separately. This means that we allow for differences in the processes underlying the decisions. This may be the case because for the initial decision, participants had time (forced delay of 3-6 sec) to monitor the options and plan ahead (and we found previously that indeed they planned ahead[9]) .In contrast later decision could be executed immediately and could have been pre-planned prior to their onset.

**d.      "Decision" inertia: We were initially a bit confused by this term since participants with "decision inertia" actually end up making more risky decisions. They rather seem to have a "commitment" or "closure" inertia because they don't want to commit to a particular outcome. A clarification early on would help.**

*Apologies for the confusion. We are now clarifying the term earlier on when it is initially mentioned emphasizing how the inertia is about making a decision to commit/stop or accept an offer.*

*Intro:*

Therefore, we predict that apathy will be linked to an increased decision inertia (**hypothesis 1**), i.e. a bias to search again and again for a new offer without considering that the appropriate strategy now would be to stop the search. One could also call this a commitment or closure inertia. Note that the hypothesis is very distinct to the more simplistic idea that apathy just occurs at the action level as a failure to act quickly, which could express itself as slower response times

**e.      Sunk-cost effects: Related to the previous point. The relation of task behavior to sunk-cost effects could be clearer early on.**

*Apologies. We are now explaining sunk cost more clearly the first time that this is mentionend in the text.*

*[Results, section ‘Hypothesis 1’]*

To understand this effect in more detail, we wanted to exclude other possible explanations for the decision inertia (supplemental figure S5) by linking measures of several of these alternative effects to apathy. First, participants might be over-optimistic about how likely they were to receive a desired outcome (see figure S2 and Supplementary Results section ‘Carrying out a choice sequence’ for how this was captured in our model). We found no evidence from either the decisions (mean=0.04, 95% CI [-0.07 – 0.14]) nor the RTs (mean=0.06, 95% CI [-0.03 – 0.16]). Second, we also excluded the possibility that accumulated costs (or ‘sunk cost fallacy’) could drive their behaviour. Specifically, this was modelled as the total cost that participants had paid so far in a trial (from the initial decision to the current one), computed as the number of previous searches (i.e. the regressor modelling ‘decision inertia’) multiplied by the cost of this trial.

**f.      Report of individual effects: When reading the results I found it initially confusing that the individual effects were reported as mean parameter estimates in a model rather than as correlations. Maybe correlations would be helpful because they allow more intuitive assessments of effect sizes.**

We agree with the reviewers that (partial) correlation values are more intuitive to interpret than regression coefficients. We initially reported regression weights for two reasons. First, for internet-based – transdiagnostic papers, this appears to be the common approach (e.g. see [10–12]). Second, for regressions predicting questionnaire responses, the regression software used (BRMS via Stan in R) allowed treating responses as ordered factors, rather than continuous (e.g. if the possible response values are 1, 2 …. 6, the model does not assume that the distance between 1 and 2 is the same as between 5 and 6). This is more complicated for correlations: in short, we want to compute partial correlations; for this, we can use the residuals of the two measures of interest (say compulsivity and pre-emptive avoidance), controlling for all other measures (clinical scores, demographics, inverse temperature) of no interest. These residuals can then be entered in a correlation, thus giving a partial correlation. It is not quite clear what to do for the ordered factors: Pearson/ Kendall correlation treat factors as numeric, while polychoric correlations can treat them as ordered factors, however, when they have been transformed to residuals, they also no longer consist of e.g. 6 categorical answers.

Based on these considerations, we have, for the discovery sample, now included figures of the correlations in the supplements (see below), and also maintained reporting of the regression coefficients in the main text.

Figure R8 (new figure S6). Partial correlations between clinical factors and behavioural measures, controlling for other clinical, demographic and general task performance measures (see methods ‘Relating questionnaires to task performance and self-reports’. Significance was assessed using non-parametric correlations (Kendall’s tau). Panels A-B correspond to regression coefficients shown figure 3A-B. Panels C-F correspond to regression coefficients shown in figure 4 A-D. Note that for correlations of clinical factors with self-report questionnaires (E and F), responses needed to be treated as continuous, while in the regression analysis they were treated as ordered factors.

*Methods [Section ‘Relating questionnaires to task performance and self-reports’]:*

To illustrate the relationship between psychiatric factors and behaviour, we also plot partial correlations of the residuals. Residuals were computed in two regressions. First the behaviour of interest was predicted as a function of all factors (i.e. psychological and control measures) listed above, apart from the one of interest (e.g. apathy). Second, the residual variance in the behaviour was correlated with the questionnaire dimension of (e.g. apathy) using non-parametric correlations (Kendall’s tau) to ensure results were not driven by outliers. For the analyses of the self-report questionnaire scores, residuals could only be computed by treating them as continuous.

**g.      "Model-free" descriptive statistics and posterior predictive checks: Some more "model-free" diagnostics would be helpful. For example, what is the average number of searches per trial? Also, posterior predictive checks for the main model variables would be needed - as well as scatter plots for the main hypotheses (and ideally individual data points or violin plots).**

*We completely agree with the reviewers about this point and apologize for this omission in the original manuscript. We have now included a figure (see answer to question 2a above; updated figure 1 E-F in paper) that shows summary plots of participants’ (model-free) behaviour, together with predictions from the model, particularly related to the measures of interest (F). In short, we find that the model captures the main aspects of participants’ behaviour: searching more, the more searches have already been done, searching less if the cost is higher. We also noticed that for the highest number of previous searches, the pattern of costs was less clear; however, it is also worth pointing out that (shown by dot size) for these bins very few data points were available across participants. We have also included correlation scatter plots (see answer to question f above).*

**3)      Analyses of questionnaires:**

**a.      4 factors: Why did the authors restrict themselves to 4 factors? How much variance do these explain?**

*The number of factors to include was determined based on a technique called Parallel Analysis [13], we apologize that we had omitted the reference to this in the results section (now added). This provides an objective (in contrast to e.g. visual inspection of the scree plot for a ‘kink’) way of determining the number of factors. To gain additional information about how stable this factor solution was, we also half split the participant sample and ran separate factor analyses on each half. The results (figure S3) show that the factor solutions obtained are very comparable. The cumulative variance explained by the four factors was 57%.*

*Results [section ‘Clinical profile’]:*

We specified four factors to extract, based on the results of parallel analysis *[13]*. These four factors (fig 4C) could be labelled ‘compulsivity’, ‘apathy’, ‘depression/anxiety’, ‘social anxiety’ and explained cumulatively 57% of variance.

**b.      Influence of "social anxiety:" Do the authors have explanations for the negative effect of social anxiety on RTs (in Figure 3B & 4B)?**

*We did not have any prior hypotheses about social anxiety, nor did we expect this particular effect to emerge, which is why we did not speculate about it further. We are mentioning it and will report it also for the confirmation sample, to give good representation of our data. The effect is particularly puzzling to us as there is no overt social component to the task. We can only speculate that potentially a heightened anxious state leads to some response speeding through faster action selection. However, the other possibility is that this effect is a false positive in our data and that it will not replicate in the confirmation sample. We will test this as an exploratory analysis in the confirmation sample.*

**4)      Various minor comments:**

**a.      The introduction is very short and mostly lists the task and the hypotheses.**

*We have now added additional clarification of the discovery/confirmation sampling procedure, more detailed descriptions of the proposed analyses and hypothesis as well as their reasoning based in this and the other reviewers comments, please see specific comments above and below and also main manuscript for fully changed introduction.*

**b.      It could be clearer why the authors use an individual differences approach. For example, another valid approach would be to test to groups of patients that differ in apathy and compulsivity (e.g., MDD v. OCD patients).**

*First, we agree that comparing groups of patients and patients and controls is obviously interesting and of value and we have made this clear in the revised manuscript. We were, however, particularly interested in the potential existence of diverse contributing factors, variation in the population even outside of the clinical range, and similarities and relationships between behavioural patterns across different traditional psychiatric dimensions. This is most easily achieved by sampling across all dimensions rather than by a group comparison approach. We have now clarified this in the text as shown further below.*

*Making categorical comparisons between patient groups and controls is an important line of research that has delivered major insights into changes in cognition and behaviour in psychological illness. The dimensional approach, nevertheless, has important strengths in that it identifies new behavioural profiles by pulling together behavioural features that co-vary which each other and it identifies patterns that naturally co-occur. , Simple questionnaire scores are important because they are based on diagnostic and clinical intuition but they may overlook co-morbidities in behavioural and cognitive patterns both within and across traditional clinical types. The reviewer is of course correct, that it is possible to design targeted questionnaires to assess specific real-life behaviours. However, often those questionnaires are nowadays validated using factor analysis and dimensionality based approaches to ensure the assumed construct actually constitutes a distinct dimension within the population rather than being questions with only face validity. There are not only theoretical reasons to believe that dimensionality based approaches might be better linked to naturally occurring variations within a population, Gillan et al., has shown that dimensionality based assessment of compulsivity can be superior to diagnostic labels in teasing apart behavioural differences linked to task measures.*

Introduction:

Making categorical comparisons between patient groups and controls is an important line of research that has delivered major insights into changes in cognition and behaviour in psychological illness. An alternative approach is to use a transdiagnostic dimensionality-based approach. We chose this approach here due to its ability to extract multiple distinct dimensions within large population data and across different questionnaires and traditional diagnostic boundaries [14,15]. Specifically, we collected questionnaires with measures covering a wide range of psychiatric symptoms and then extracted symptom dimensions cutting across traditional disorder boundaries. This is particularly relevant for three reasons. First, many disorders, such as obsessive-compulsive disorder (OCD), have co-morbidity rates as high as 90% [7]. Secondly, it is well known that some dimensions, are shared across many disorders [8]. Third, recent work [6] suggests that dimensions cutting across disorders relate more strongly to behavioural deficits than diagnostic categories.

c.      How long is the experiment? How do the authors make sure that the online participants don't interrupt the experiment? We're aware that Prolific includes a 'Maximum time allowed', but we assume that many people won't be aware of this and therefore might be concerned that participants take long breaks between parts of the experiment (e.g., take a long break between the behavioral task and the questionnaires)

*Participants took on average about 84 minutes to complete the entire experiment (see table R6 below). In retrospect, we thought this was too long for an online study, which is why we have shortened the task and removed questionnaires for the replication sample (see table R5 above that details differences between discovery and confirmation samples). We did not penalize participants for interrupting the experiment as it was not a learning task, but a decision making task, in which each round was independent of other rounds, so we would expect taking breaks to have less of a detrimental effect on behaviour than in a learning task. However, we excluded trials from RT analysis with particularly long RTs. In terms of participants being aware about the ‘time out’, results below in table R6 suggest that they probably were aware of it: participants who we excluded based on problematic responses to the questionnaires had taken longer on average to complete training and task and then responded much faster to the questionnaires, we presume because they were worried about the time out – based on initial pilot data we advertised the experiment as taking less than 80 min (though we did pay participants if they took longer than the estimated time).*

Table R6 (Table 1 in main text now): Demographics and duration of experiment for participants that we included or excluded from all analyses based on questionnaire and task performance criteria (see Methods for details).

**d.      The authors exclude persons with too little variance in their answers, which is completely fine. However, since so many questionnaires are available, it could be interesting to explore the questionnaire variables of these "excluded" participants. In other words: is there anything interesting/unusual about the people who had little variance?**

*As explained in response to c. above, we are worried that in the discovery sample, participants were excluded from questionnaires for unfortunate reasons: being rushed after taking longer than expected to complete training and task; additionally, we did not highlight to participants the importance of the questionnaire part of the study. We will address this in the confirmation sample by: ensuring the time advertised is appropriate, including information about importance of questionnaires, giving participants warning while completing questionnaires if they are completed too quickly and including repeat questions (and making participants aware of this) to exclude questionnaire data based on this rather than response speed. As a result of these changes, we expect that we can be more certain that even responses of participants excluded based on their questionnaire responses meaningfully reflect something about participants’ inner experience, rather than spurious effects introduced by our experimental design. We will then be able to analyse behavioural patterns for both participants who had valid and invalid questionnaire responses.*

*Revised and simplified questionnaire exclusion criteria, Methods [section ‘Participant exclusion criteria’]*

Exclusion based on questionnaire responses:

1) Not completing all psychiatric questionnaires

2) Five questions will be repeated. If a participant’s responses are different by two or more points on average, they will be excluded.

**e.      In the power analyses presented in Figure 5, how can power decrease with larger sample size (e.g., 'Compulsivity x Chasing' has ~96% power at 450 participants, but only ~87% power at 550 participants)? Is this an artefact of the randomness in the simulations? If so, would more simulated runs be necessary to avoid this?**

*We are very grateful for the reviewers picking up on this. We have now re-run the power analysis with 500 samples each. And in fact the curves are now much smoother. Based on this we have now increased the sample size for the replication sample to 750.*

*Figure R6 (Updated Figure 5). Power analysis.* Power increases with sample size. To reach 95% power in the analysis with lowest power (i.e. analysis of the impact of apathy on over-perseverance measured in the participants’ decisions), we selected 750 participants for the confirmation sample.

**f.      Although we appreciate the authors' attempt to make their research question especially relevant to current debates, it seems unlikely that sequential decision-making has become dramatically more common for animals and humans since 01.01.2000 (see 2nd sentence in introduction and their explicit emphasis on the 21st century). It should also be added that humans are animals, so that distinction seems somewhat redundant**

*We apologies for the misunderstanding. Of course, we didn’t not want to argue that sequential decision making has become more common or that humans and animals differ. In fact, we believe the exact opposite, i.e. that humans AND animals have always faced a huge number of sequential decisions. However, most research in decision making has neglected this fact (except a few notable exceptions), which is what we were trying to point out.*

*We have changed the manuscript adding the paragraph below to reflect this.*

*Introduction:*

Real life decisions often involve planning a sequence of decisions and then carrying it out while monitoring the appropriateness of the current behavior. Such sequential decision making problems, rather than single event decisions, often characterize the decision making problems that have to be solved by foraging animals and, equally, by humans*. While sequential decisions are ubiquitous in nature, very little attention has been paid to cognitive models of decision making that take sequential context and planning into account. This is an important issue, as many decision problems, and the cognitive mechanisms needed to deal with them, are unique to sequential decisions* [16–18]*.*

**g.      The authors state multiple times that they want to get more ecological validity and that sequential decision-making is more realistic and that the dimensional approach is more realistic, but the last point is not so obvious: why exactly is the dimensional approach more realistic than just the questionnaires? Isn't the whole point of a well-designed questionnaire to learn something about someone that can help make predictions about their real-life behavior?**

*The reviewers touch on an important point here: it is worthwhile considering what to contrast the dimensional approach with. We could either contrast it with using questionnaires or we could contrast it with an approach using patients that are put into categories according to diagnoses.*

*We agree with the reviewers that in theory, the dimensional approach is not (much) better than the questionnaire approach: ideally questionnaires should be structured so that they already just measure dimensions. In practice however, this is not necessarily the case. For example, we find (and recently Gillan et al. published the same finding* [6]*) that from the obsessive compulsive inventory almost all scales load onto the compulsivity factor, however crucially the scale of obsessive thinking loads onto the depression factor. So, if instead of using the dimensional approach, we had formed summed scores simply across each questionnaire, this would have been lost. However, we agree with the reviewers that this is maybe a minor argument in favour of a dimensional approach and ultimately one would hope that through this new transdiagnostic work done by more labs, better questionnaires will ultimately emerge.*

*What we would argue more strongly is that the dimensional approach has something to add beyond the classic ‘controls vs. patients’ approach. As we describe above, the dimensional approach is more realistic in that it allows for comorbidities between disorders and dimensions being shared across disorders. However, of course, the dimensional approach complements rather than replaces the control vs. patient approach as there are also important advantages to control vs patient sampling.*

*See the introduction changes that we have included above that hopefully reflect these considerations more clearly now.*

**References**

1.	Anderson M, Magruder J. Split-Sample Strategies for Avoiding False Discoveries. Cambridge, MA: National Bureau of Economic Research; 2017 Jun p. w23544. Report No.: w23544. doi:10.3386/w23544

2.	Fafchamps M, Labonne J. Using Split Samples to Improve Inference on Causal Effects. Polit Anal. 2017;25: 465–482. doi:10.1017/pan.2017.22

3.	Arditte KA, Çek D, Shaw AM, Timpano KR. The importance of assessing clinical phenomena in Mechanical Turk research. Psychol Assess. 2016;28: 684–691. doi:10.1037/pas0000217

4.	Seow TXF, Gillan CM. Transdiagnostic Phenotyping Reveals a Host of Metacognitive Deficits Implicated in Compulsivity. Sci Rep. 2020;10: 1–11. doi:10.1038/s41598-020-59646-4

5.	Ophir Y, Sisso I, Asterhan CSC, Tikochinski R, Reichart R. The Turker Blues: Hidden Factors Behind Increased Depression Rates Among Amazon’s Mechanical Turkers. Clin Psychol Sci. 2020;8: 65–83. doi:10.1177/2167702619865973

6.	Gillan CM, Kalanthroff E, Evans M, Weingarden HM, Jacoby RJ, Gershkovich M, et al. Comparison of the Association Between Goal-Directed Planning and Self-reported Compulsivity vs Obsessive-Compulsive Disorder Diagnosis. JAMA Psychiatry. 2020;77: 77–85. doi:10.1001/jamapsychiatry.2019.2998

7.	Ruscio AM, Stein DJ, Chiu WT, Kessler RC. The epidemiology of obsessive-compulsive disorder in the National Comorbidity Survey Replication. Mol Psychiatry. 2010;15: 53–63. doi:10.1038/mp.2008.94

8.	Husain M, Roiser JP. Neuroscience of apathy and anhedonia: a transdiagnostic approach. Nat Rev Neurosci. 2018;19: 470–484. doi:10.1038/s41583-018-0029-9

9.	Kolling N, Scholl J, Chekroud A, Trier HA, Rushworth MF. Prospection, perseverance, and insight in sequential behavior. Neuron. 2018;99: 1069–1082.

10.	Gillan CM, Kosinski M, Whelan R, Phelps EA, Daw ND. Characterizing a psychiatric symptom dimension related to deficits in goal-directed control. Elife. 2016;5: e11305.

11.	Rouault M, Seow T, Gillan CM, Fleming SM. Psychiatric symptom dimensions are associated with dissociable shifts in metacognition but not task performance. Biol Psychiatry. 2018;84: 443–451.

12.	Wise T, Dolan RJ. Associations between aversive learning processes and transdiagnostic psychiatric symptoms revealed by large-scale phenotyping. bioRxiv. 2020; 843045. doi:10.1101/843045

13.	Hayton JC, Allen DG, Scarpello V. Factor Retention Decisions in Exploratory Factor Analysis: a Tutorial on Parallel Analysis. Organ Res Methods. 2004;7: 191–205. doi:10.1177/1094428104263675

14.	Stephan KE, Bach DR, Fletcher PC, Flint J, Frank MJ, Friston KJ, et al. Charting the landscape of priority problems in psychiatry, part 1: classification and diagnosis. Lancet Psychiatry. 2016;3: 77–83. doi:10.1016/S2215-0366(15)00361-2

15.	Cuthbert BN. The RDoC framework: facilitating transition from ICD/DSM to dimensional approaches that integrate neuroscience and psychopathology. World Psychiatry. 2014;13: 28–35. doi:10.1002/wps.20087

16.	Kolling N, Wittmann M, Rushworth MFS. Multiple Neural Mechanisms of Decision Making and Their Competition under Changing Risk Pressure. Neuron. 2014;81: 1190–1202. doi:10.1016/j.neuron.2014.01.033

17.	McNamara JM, Trimmer PC. Sequential choices using signal detection theory can reverse classical predictions. Behav Ecol. 2019;30: 16–19. doi:10.1093/beheco/ary132

18.	Korn CW, Bach DR. Maintaining Homeostasis by Decision-Making. PLOS Comput Biol. 2015;11: e1004301. doi:10.1371/journal.pcbi.1004301
