# Supplementary material for: The effect of apathy and compulsivity on planning and stopping in sequential decision-making
Source: PLoS Biol. 2022 Mar 31;20(3):e3001566. doi: 10.1371/journal.pbio.3001566 (PMC8970514; doi:10.1371/journal.pbio.3001566)
Supplement: Attachment — Submitted filename: Notes to the reviewers_250521.docx [file pbio.3001566.s024.docx]

25^th^ May 2021

**PBIOLOGY-D-19-03235R2: Should I stick with it or move on? The effect of apathy and compulsivity on planning and stopping in sequential decision making**

Dear reviewers,

To make reviewing of the manuscript easier we wanted to highlight several features of the manuscript.

In particular, we made some minor changes in the methods compared to the pre-registration documents. They are clearly highlighted and fell into the following categories:

- Simplification of the language and explanations for the analyses. In particular, we added some more detail, when we believed it was lacking, to facilitate better reproducibility of the analysis.
- We removed all the sections referring to the pre-registered RT analysis as those were based on an indexing error in the discovery sample. We clearly explain this error in several places in the manuscript.
- We added clearly marked exploratory sections to explain our new exploratory results
- We expanded how we confirm our factor analysis and added some more details about how we implemented it.

All changes in the methods are clearly marked as such and highlighted in the manuscript in red.

Beyond this, we added some supplemental information as we thought it might be helpful for the readers and because it gives a more comprehensive picture including suggestions made by the reviewers such as medication status.

Abstract, results and discussion have also changed as they reflect the results in the confirmation sample rather than the original discovery sample. We left the introduction itself unchanged as it sets out the rationale which remained the same.

Yours Sincerely,

Jacqueline Scholl	Matthew Rushworth	Nils Kolling
