# Supplementary material for: The effect of apathy and compulsivity on planning and stopping in sequential decision-making
Source: PLoS Biol. 2022 Mar 31;20(3):e3001566. doi: 10.1371/journal.pbio.3001566 (PMC8970514; doi:10.1371/journal.pbio.3001566)
Supplement: Attachment — Submitted filename: Reply to the reviewers 170122.docx [file pbio.3001566.s026.docx]

Comment to the editor:

In addition to answering the reviewers comments we have also added data file references to the figures of the manuscript and supplements and uploaded the data to the osf pre-registration. We have also prepared the necessary data files for all the PLOS Biology figures so that you can link those to the final manuscript.

Reply to the reviewers PBIOLOGY-D-19-03235:

Reviewer #2:
[identifies himself as Jiaxiang Zhang]

I have reviewed the Stage 1 report of this study. It is great to see the confirmatory results in the current report. In this manuscript, the intro, rationale, and hypotheses are the same as the stage 1 protocol, making it a high-quality stage 2 submission. The authors did a great job in adhering to original experimental procedures and analyses plans. In the case of potential inconsistency (e.g., factor analyses), the authors provided convincing evidence to stick with the original analysis plan. Overall, beyond the obvious interest to the decision-making/cognitive psychology community, I believe this study will set a nice example on how to combine a novel experimental paradigm with rigorous pre-registered analyses. Below are just a few minor comments.

1. Table 3. Although exploratory analyses are clearly marked as they are, I wonder if the authors could unpack the results in Intro/Discussion a bit more, as they can be as useful as confirmatory results for future studies. What do they mean, what can we learn from them, or how can they be extended in future studies? For example, cost is not a reliable predictor (which I think is the same predictor in hypothesis 2A?) of compulsivity, but "chasing" is. There are however other task-based measures that, together, yield a better predictive model for compulsivity. Does this mean trait measure of compulsivity is associated with multifaceted behaviour and meta-cognitive processes, instead of specific correlations highlighted by the main hypotheses? Similarly, could the authors comment on those task-based regressors predicting age/gender? Are they in line with the current literature?

We agree with the reviewer and added the following text to the discussion to highlight the possibility within the additional exploratory analysis:” Overall, the analysis highlights the possibility that links between metacognition, task behaviour and clinical dimensions can be multi-facetted i.e. not map one-to-one with each other. In our data this was the case for compulsivity, which was best predicted by a mixture of metacognitive measures (e.g. self-reported chasing) and behavioural measures (such as choice stochasticity). Importantly, to find and validate such multi-facetted relationships a discovery-confirmation sample approach is particularly valuable as it allows for the initial identification of the combination of the relevant factors in the discovery sample and a test of these factors in the confirmation sample. While we had no prior hypotheses about age and gender, the mixtures of effects are in line with previous studies showing differences in risk taking between male and female participants [70] and changes in decision making with age [71]. However, again, they highlight how with complex traits, a larger set of behavioural and metacognitive changes can emerge in more complex tasks.“ [Page 22]

2. Page 22. "95% % CI_lower:1.081, hypothesis 2C …" Do the authors mean hypothesis 2B here? As 2C refers to the hypothesis on RT.

Yes, corrected.

3. Table 4. I suggest having a clear outcome report for each sub hypothesis. I.e., H1B/H2C is n/a nor not tested. H2A is not supported but H2B is confirmed (avoiding the ambiguous "partially confirmed").

We agree and have made the suggested change. 

4. Page 44 "Step 3: We applied the models from step to the confirmation sample" --> "from step 2" ?

Corrected.

[re PLOS' data availability policy]
The authors claimed that the data will be made available after acceptance. As a result, data availability/time stamps cannot be reviewed at this stage.


Reviewer #3:
[identifies himself as Christoph W. Korn]

In my view, Scholl and colleagues have provided a very comprehensive and detailed Stage 2 manuscript. Given this complexity, it took me longer than expected to carefully assess the criteria listed for Stage 2 manuscripts. I did not find any flaws. The few deviations from Stage 1 are explained (e.g., the RT analyses). In my view, only the last criterion is not yet fulfilled since the data is not yet freely accessible. 

Below, I list a few minor suggestions for improving the clarity of the manuscript.

1. Figure 1F: Why does the model fit get worse with increasing numbers of previous searches? Is this simply because there are less data points available? Is there any particular reason for the "peak" at "6 previous searches" for "cost 15?"

We agree with the reviewer that the likely cause of variability in fits is due to differences in data points. To illustrate this, we had varied the dot size in the figure as a function of data points. We are not aware of any specific feature of our task or schedule that would produce a peak at 6 searches. It is possible that it is due to the fact that committing more than 6 searches, particularly in the high cost condition is very unlikely, making fits worse or that participants might irrationally “give up” searching because of the costs incurred, leading to a reduction of % search in that particular condition.

2. In some places, the distinction between self-reported "pre-emptive avoidance" and the parameter 'AvgProspVChange' is a bit confusing.
a. At first, it sounds as if the authors just measured a self-report but it later becomes clear that they also have decision variable.

b. It was unclear to me why the authors did not look into 'AvgProspVChange' (see footnote e). This could be an interesting exploratory analysis.

Footnote e related to an analysis we had run in a previous paper about moderation between decision inertia and pre-emptive avoidance (as measured by AvgProspVChange) by awareness of decision inertia. However, this analysis does not directly relate to the questions about clinical differences, which is why we didn’t run it here. We did look at the main effect of AvgProspVChange in first decisions on factors such as compulsivity, which was not significant in the discovery sample. In fact, all significant associations in the discovery sample are part of the exploratory analysis (Table 3). The lack of an effect of AvgProspVChange in the presence of self-reported effects, could be because the regressor might be an incomplete measure of actual pre-emptive behaviours or because metacognitive beliefs are not completely aligned with participants actual behaviour.

To make this clear, we have made changes to the discussion:

“While we find that compulsivity is linked to increased self-reported pre-emptive avoidance, there was no such link to a corresponding behavioural measure aimed at this [Table S7]. This could suggest either that participants only subjectively perceive a change in their behaviour, which is in fact unchanged. Or it could suggest that the behavioural measure does not capture the full range of behaviours participants might engage in order to pre-emptively avoid but which they consider when they self-report.” [Page 22]

c. In the abstract, the authors write: "In addition, this awareness allowed them to report pre-emptively avoiding situations related to the bias." The question that comes to mind immediately is: "Did participants actually pre-emptively avoid those situations?"

Related to our answer to 2.b, all significant associations in the discovery sample are part of the exploratory analysis (Table 3). The lack of an effect of AvgProspVChange in the presence of self-reported effects, could be because the regressor might be an incomplete measure of actual pre-emptive behaviours or because metacognitive beliefs are not completely aligned with participants actual behaviour. This means we are cannot with certainty say that participants did not pre-emptively avoid behaviourally either way i.e. whether belief and behaviour are misaligned, just that it wasn’t reflected in our behavioural surrogate measure for pre-emption.

We have now added the following sentence to the abstract to make this clear:

“*In addition, this awareness made them report pre-emptively avoiding situations related to the bias. However, we found no evidence of them actually pre-empting more in the task, which might mean a misalignment of their metacognitive beliefs or that our behavioural measures were incomplete.” [Page 1]*

3. A few details could be mentioned briefly or explained more clearly in the results (and not "just" in the methods).
a. How high are the correlations between decision variables? (below .7).

We have now added the average correlations to the supplements (see below):

**“**The average correlations (Pearson’s r) between regressors used in the reaction time regression and the decision-making models are at or below 0.7 for all variables for initial decisions (Bi) and later (Bii).”

b. The distinction in the analyses between the first and the later searches could be stressed a bit more in the results.

We have now added “We split the behavioural data into initial and later searches to be able to test effects of prospection at the first decision separately from sequential decision effects such as perseverance. “[Page 6] to address the reviewers concern.

c. It could simply be stated that all results from the discovery sample are reported in the Supplemental Material and links to the corresponding figures/tables could be given.

We now added “Results of the discovery sample can be seen in the supplementary material and the pre-registration (<https://osf.io/dfg2u/)>.” “[Page 6].

4. Style:
a. Figure 3B (and others): The labels are difficult to read. Usually, the columns are ordered in the same way as the rows and therefore the main diagonal runs from upper left to lower right. Maybe, the easiest way is to just show the lower (or upper) triangular part of the matrix.

We agree and have made the y-labels clearer to read. We also happy to remove them completely, as they are somewhat redundant.

b. In the abstract (and also later), the term "perceived insensitivity" sounds ambiguous. "Self-reported" might be a better word.

We agree and have changed the word throughout.

c. In Table 4, "partially confirmed" could be replaced by a statement specifying the difference between the discovery and the confirmation samples.

Based on this and reviewer 2 we have now changed the table to state confirmation for each sub hypothesis separately. We hope that makes the differences clear.

d. Figure 1: The figure is quite complex and the caption is very long. Splitting this up could help.

We have tried the two elements of the figure clearer by adding a separation.

e. There are some typos or omissions: e.g., "Batchelor," "large set regressor-to-factor relationships"

Many thanks for pointing those out, as those mistakes can easily stay unnoticed otherwise! Specifically, apologies for using the british spelling of Bachelor. We have corrected the omission of “of”

f. The title for the supplements is different.

Many thanks for catching that we had failed to update the heading for the supplement !
